# A *Phytophthora sojae* cytoplasmic effector mediates disease resistance and abiotic stress tolerance in *Nicotiana benthamiana*

**DOI:** 10.1038/srep10837

**Published:** 2015-06-03

**Authors:** Meixiang Zhang, Nasir Ahmed Rajput, Danyu Shen, Peng Sun, Wentao Zeng, Tingli Liu, Joseph Juma Mafurah, Daolong Dou

**Affiliations:** 1 Department of Plant Pathology, Nanjing Agricultural University, Nanjing, China; 2 Department of Plant Pathology, Sindh Agriculture University, Tandojam, Pakistan

## Abstract

Each oomycete pathogen encodes a large number of effectors. Some effectors can be used in crop disease resistance breeding, such as to accelerate *R* gene cloning and utilisation. Since cytoplasmic effectors may cause acute physiological changes in host cells at very low concentrations, we assume that some of these effectors can serve as functional genes for transgenic plants. Here, we generated transgenic *Nicotiana benthamiana* plants that express a *Phytophthora sojae* CRN (crinkling and necrosis) effector, *PsCRN115*. We showed that its expression did not significantly affect the growth and development of *N. benthamiana*, but significantly improved disease resistance and tolerance to salt and drought stresses. Furthermore, we found that expression of heat-shock-protein and cytochrome-P450 encoding genes were unregulated in *PsCRN115*-transgenic *N. benthamiana* based on digital gene expression profiling analyses, suggesting the increased plant defence may be achieved by upregulation of these stress-related genes in transgenic plants. Thus, *PsCRN115* may be used to improve plant tolerance to biotic and abiotic stresses.

Effectors are molecules secreted by pathogens that manipulate host cell function, thereby facilitating infection or triggering defence responses^1^. The dual functions of effectors have been extensively reported in many plant-microbial pathosystems^2^. In the presence of the corresponding resistance (R) protein, the effector is recognised as an avirulence (AVR) factor, resulting in a defence response often associated with a hypersensitive response (HR) that restricts the spread of pathogens from the infection site. It is believed that effectors contribute to virulence in a susceptible host. Indeed, the virulence functions of many effectors have been characterised^3^,^4^.

Plant-pathogenic oomycetes cause devastating diseases on many different host plants and have a huge impact on agriculture. For example, *Phytophthora infestans* is responsible for the Irish potato famine; *P. sojae* causes root and stem rot of soybean (*Glycine max*), which is one of the most important pathogens of soybean^5^. Bioinformatic analyses of the genome sequences of several oomycete pathogens have identified a large number of candidate effector proteins^6^,^7^. Among them, RxLR and CRN effectors are two major classes of well-studied cytoplasmic effectors. RxLR effectors are defined by a conserved motif in the N-terminus, termed RxLR (for Arg, any amino acid, Leu, Arg), which mediates translocation of effectors into host cells^8^,^9^. To date, nearly all avirulence genes identified from oomycete plant pathogens belong to RxLR effectors^10^. Virulence functions of several RxLR effectors have been characterised, including the avirulent proteins PiAvr3a, PiAvrblb2, PsAvr3b, HaATR1, and HaATR13^10^. Interestingly, recent work on *P. capsici* correlated non-host resistance (NHR) in a range of *Nicotiana* species with HR elicited by a single RxLR effector, PcAvr3a-like^11^.

CRNs were first identified in *P. infestans*, and their transient expression resulted in a leaf-crinkling and cell-death phenotype in plants^12^. The CRN effector family shows extensive expansion in all sequenced *Phytophthora* species. Analogous to the RxLR effectors, the N-terminus of CRN contains a conserved FLAK (for Phe, Leu, Ala, Lys) motif required for effector translocation^13^. In contrast to the N-terminus, CRN C-terminal domains have high levels of variation and control virulence. Our previous studies revealed a role of some CRN C-termini towards *P. sojae* virulence on soybean^14^. It has also been shown that the *P. infestans* CRN8 C-terminus contains a kinase-like domain and exhibits kinase activity, suggestive of a role in modification of host cell signalling pathways during infection^15^. Recent studies showed that the expression of CRN effectors rarely leads to cell death^16^,^17^. Interestingly, we showed previously that many CRNs from *P. sojae* suppress cell death induced by PAMPs or other elicitors, suggestive of diverse activities^16^. However, only one CRN from *P. capsici* has a positive effect on pathogen virulence in a screening assay^17^, suggesting that CRNs play a more subtle role in plant-pathogen interactions than believed previously. The majority of CRN effectors are localised to plant nuclei, indicating that CRNs target and perturb host nuclear processes to exert effector activity^17^. However, the functions and molecular mechanisms of most CRN effectors remain unclear.

Plants are constantly exposed to a variety of biotic (i.e. pathogen infection and insect herbivory) and abiotic (i.e. high temperature, drought, and high salinity) stresses throughout their life cycles. These stresses negatively impact plant growth and development, and cause considerable losses in crop yield worldwide. Many attempts have been made to improve resistance to pathogens and increase tolerance to abiotic stress. One commonly used strategy is to overexpress plant genes that are induced by biotic or abiotic stresses, such as mitogen-activated protein kinases^18^,^19^, transcriptional factors^20^, and disease-related genes^21^. Another important strategy is to introduce heterologous genes that confer resistance to pathogens or abiotic stress^22^,^23^.

It has been shown that the expression of pathogen-derived elicitors induces plant immune responses and improves plant resistance to pathogens or insects^24^,^25^,^26^. Elongation factor (EF-Tu), a highly conserved elicitor in bacteria, can induce defence responses in plants. The N-terminal 18 amino acids of EF-Tu can trigger plant immune responses^24^. Flagellin is another well-characterised elicitor in bacteria. Expression of a flagellin gene from the bacterial pathogen *Acidovorax avenae* in rice enhances disease resistance against the fungal rice blast pathogen *Magnaporthe grisea*^27^. However, transgenic plants showed small chlorotic spots on leaves, indicating that the constitutive activation of flagellin-mediated immune responses causes detrimental effects on plants. Harpins (Hrps), another class of bacterial-pathogen-derived elicitors, can also be used to improve plant defence. Interestingly, Hrp protein treatment not only improves plant tolerance to drought stress^28^ but also promotes plant root growth^29^. Expression of a functional fragment of harpin protein Hpa1 increased resistance to fungal pathogens and insects^30^,^31^. Interestingly, although Hpa_10-42_ expression represses root development, it enhances the growth of aerial parts of plants^30^.

PsCRN115 and PsCRN63 were previously identified from *P. sojae*, and share high sequence similarity, but exhibit contrasting activities on host plants. PsCRN63 triggers cell death, while PsCRN115 suppresses cell death. Silencing of *PsCRN115* and *PsCRN63* simultaneously reduces *P. sojae* virulence^14^; however, the roles of PsCRN115 and PsCRN63 during pathogen infection remain unclear. To evaluate the effects of *PsCRN115* expression on plant defence, we transferred *PsCRN115* into the model plant *Nicotiana benthamiana*. We found that expression of *PsCRN115* did not lead to obvious developmental changes in *N. benthamiana*. Unexpectedly, *PsCRN115* expression increased plant resistance to *Phytophthora* pathogens and improved plant tolerance to salt and drought stresses. Upregulation of heat-shock-protein (HSP) and cytochrome-P450 genes may contribute to biotic and abiotic stress resistance in *PsCRN115*-transgenic *N. benthamiana*. Our results suggest that the oomycete effectors can be used to improve plant tolerance to biotic and abiotic stresses.

## Results

### *PsCRN115* expression does not significantly affect the growth and development of *Nicotiana benthamiana*

**Figure 1:**
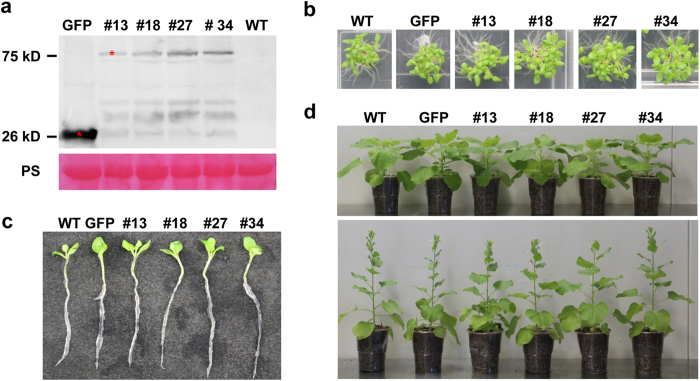
Characterisation of *PsCRN115*-transgenic *Nicotiana benthamiana*. (**a**) Western blot analysis of total proteins from leaf extracts of transgenic *N. benthamiana* lines of T3 generation. Total proteins were extracted from 40-day-old leaf tissues of *PsCRN115* transgenic *N. benthamiana* lines (#13, #18, #27 and #34) and the *GFP* transgenic *N. benthamiana* line (GFP). The monoclonal antibodies against GFP were used for protein expression detection. PS is Ponceau stain. (**b**) Seed germination of *N. benthamiana* plants on MS medium 12 days after sowing. (**c**) The post-germination growth of transgenic seedlings on MS medium. The root length (10 seedlings for each transgenic line) was recorded 14 days after sowing. (**d**) The developmental phenotypes of transgenic plants at 7 (above) and 10 weeks (below) post-germination. WT, wild-type *N. benthamiana* used as recipient; GFP, *GFP*-transgenic *N. benthamiana*; #13, a *PsCRN115*-transgenic line used as an example.

We showed previously that silencing of *PsCRN63* and *PsCRN115* jointly reduced *P. sojae* virulence^14^. To further explore the function of *PsCRN115*, we generated a *GFP:PsCRN115* fusion construct driven by the CaMV 35S promoter, and introduced it into *N. benthamiana* by *Agrobacterium*-mediated leaf disc transformation^32^. A total of 34 independent *PsCRN115*-expressing lines were obtained using kanamycin resistance selection. Transgene integration and expression was further confirmed based on genomic PCR and RT-PCR (Supplementary Fig. S1). Furthermore, Western blot was conducted to confirm that GFP:PsCRN115 fusion protein was properly expressed in *N. benthamiana* at the expected size in T1 transgenic plants (Supplementary Fig. S1). Four representative lines (#13, #18, #27 and #34) were selected for further characterisation because *PsCRN115* was correctly expressed. T3 progeny plants of these lines were confirmed by Western blot (Fig. 1a) and used for further phenotypic characterisation.

It was previously shown that *PsCRN115* is localised to the plant nucleus using *Agrobacterium*-mediated transient expression^14^. We confirmed the nuclear localisation of PsCRN115 in stable transgenic lines under normal and stress conditions (Supplementary Fig. S2). This result suggested that PsCRN115 targets plant nuclei to exert its biological function. To determine whether the transgenes affect plant development, we observed growth and development of the transgenic lines at different stages. Germination rate of the *PsCRN115*-transgenic lines were similar to the wild-type and *GFP* lines 12 d after sowing on MS plates (Fig. 1b). At the same time, no significant differences in root lengths (Fig. 1c) and no visible morphological changes were observed in transgenic plants compared to wild-type and *GFP* lines (Fig. 1d).

CRNs are conserved in oomycete pathogens, and could be classified as pathogen-associated molecular patterns (PAMPs)^33^. To preliminarily determine whether PsCRN115 functions as a PAMP in *N. benthamiana*, callose deposits were detected in *PsCRN115*-transgenic lines. Callose deposits were clearly observed in a positive control for staining in which wild type plants were treated with a well-known PAMP molecule flg22. However, no signals of callose deposits were detected in wild type, *GFP*- and *PsCRN115*-transgenic lines when flg22 was absent (Supplementary Fig. S3). Thus, we suggest that expression of *PsCRN115* does not cause visible reinforcement of cell wall and not significantly affect the development of transgenic *N. benthamiana* under normal growth conditions.

### *PsCRN115* expression suppresses cell death in *N. benthamiana*

**Figure 2:**
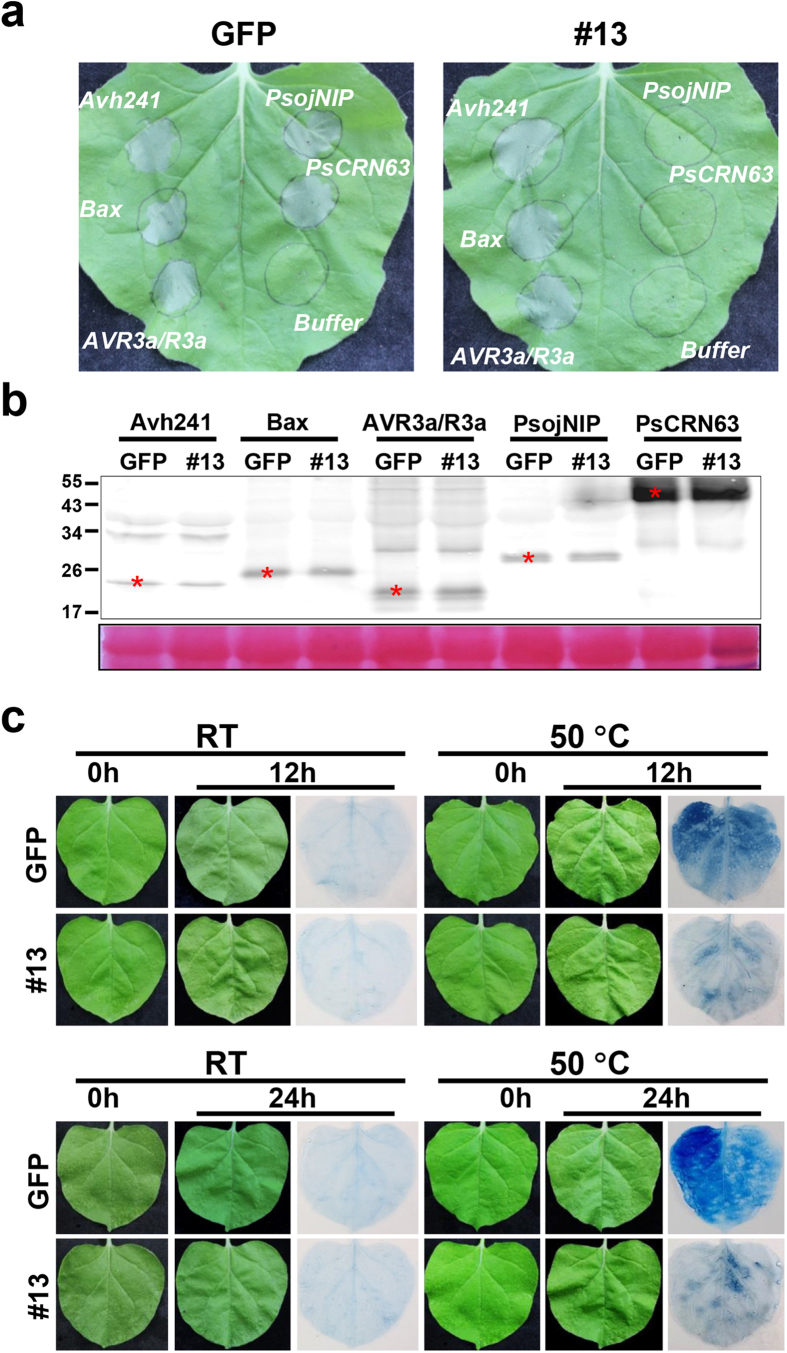
Suppression of cell death in *PsCRN115*-expressing plants. (**a**) Suppression of elicitor-triggered cell death in *PsCRN115*-transgenic *N. benthamiana*. The leaves were infiltrated with *Agrobacterium* GV3101 cells containing a PVX vector carrying *PsAvh241*, *Bax*, *Avr3a*/*R3a*, *PsojNIP* or *PsCRN63*. The photographs were taken 5 d after infiltration. (**b**) Confirmation of protein expression of the cell-death elicitors using Western blot analysis. Mouse monoclonal antibodies against the HA-epitope tag were used to detect expression of the cell-death elicitors fused to HA tags. (**c**) Heat-induced programmed cell death in transgenic *N. benthamiana*. Leaves of *GFP*- and *PsCRN115*-transgenic plants were detached and floated on water at room temperature or 50 °C for 40 min in the dark. The photographs were taken at 0, 12 and 24 h after treatment. The treated leaves were stained with trypan blue to visualise cell death. The results were reproducible in each transgenic line in at least three replicates and #13 was shown as an example.

We showed previously that PsCRN115 suppresses plant cell death triggered by PsCRN63 and PsojNIP by *Agrobacterium*-mediated transient expression^14^. To determine whether GFP:PsCRN115 fusion affects the function of PsCRN115, we conducted the cell-death-suppression assay in *PsCRN115*-transgenic lines. Cell death triggered by PsCRN63 and PsojNIP was suppressed in *PsCRN115*-expressing plants. However, cell death triggered by other elicitors was not (Fig. 2a), which was consistent with previous results based on transient expression assay^14^. This result indicated that the GFP:PsCRN115 fusion does not affect the function of PsCRN115. To determine whether the cell-death elicitor proteins were unstable in transgenic plants, Western blot analysis was conducted. The results showed that the cell death inducers were equally expressed in *PsCRN115*-transgenic plants compared to *GFP* lines (Fig. 2b), indicating that cell death suppression was caused by *PsCRN115* expression in *N. benthamiana*.

Since PsCRN115 suppresses cell death triggered by specific elicitors, we explored whether it also suppressed programmed cell death (PCD) induced by other cell‐death‐inducing stress. Exposure to heat shock can lead to PCD in a number of species, including *Arabidopsis* and tobacco^34^. The detached leaves were floated on water at high temperature (50 °C) for 40 min and transferred to room temperature and incubated for 12 hours or 24 hours. All the *PsCRN115*‐transgenic line showed reduced PCD compared to control plants as determined by trypan blue staining (Fig. 2c). Thus, PsCRN115 may protect plant cells from PCD induced by heat‐shock stress and pathogen elicitors.

### *PsCRN115* expression enhances plant resistance to *Phytophthora* pathogens

**Figure 3:**
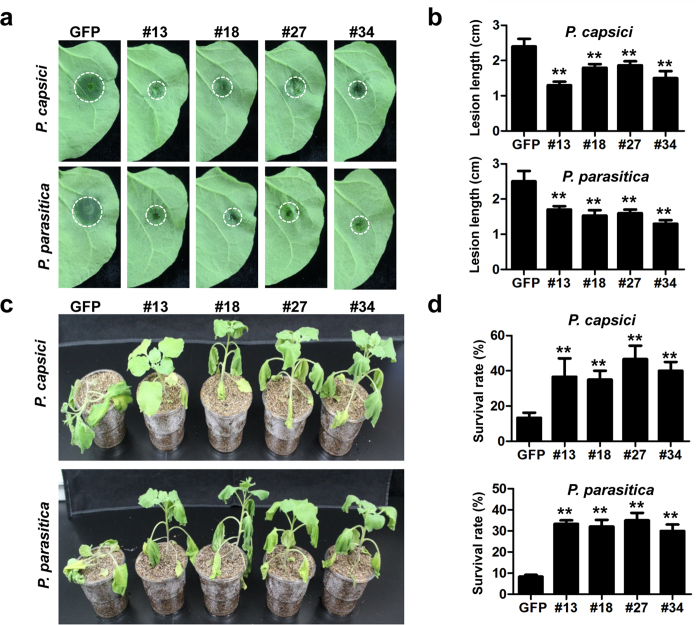
Enhanced resistance to *Phytophthora pathogens* in *PsCRN115*-transgenic plants. (**a**) Leaf phenotypes upon *Phytophthora* infection. Detached leaves of *GFP*- and *PsCRN115*-transgenic plants were inoculated with *P. capsici* or *P. parasitica* zoospores. The representative photographs were taken at 36 hpi. (**b**) Average lesion diameters of the inoculated leaves. Error bars represent standard errors calculated from at least 10 independent biological replicates. Asterisks indicate significant differences determined using Dunnett’s test (P < 0.01). (**c**) Phenotypes of the inoculated plants. A total of 5 mL of *P. capsici* or *P. parasitica* zoospores (100 μL^−1^) were dipped into the soils. The photographs were taken at 4 dpi. (**d**) Survival rates of the inoculated plants. The rates were measured at 4 dpi. The experiments were repeated four times with similar results. A total of 20 plants for each line were used for inoculation assays. Bars represent the standard errors. Different letters above bars indicate statistical significance (**, P < 0.01, Dunnett’s test).

Since PCD plays a key role in plant immunity^35^, we evaluated the effects of *PsCRN115* expression on plant resistance. We previously showed that transient expression of *PsCRN115* increased plant resistance to *P. capsici*^36^,^37^. Here we challenged the stable *PsCRN115*-transgenic plants with two oomycete pathogens (*P. capsici* and *P. parasitica*) using two methods. Detached leaves of transgenic plants were first inoculated with *Phytophthora* zoospores. All inoculated leaves showed water-soaked lesions at 36 hours post-inoculation (hpi). However, the lesion diameters were significantly smaller in *PsCRN115*-transgenic lines compared to *GFP* lines (Fig. 3a, b). We then inoculated the transgenic plants with zoospores using the root-dip inoculation method. Although both *PsCRN115*- and *GFP*-transgenic plants showed symptoms of wilting and stunting (Fig. 3c), the *PsCRN115*-expressing plants showed reduced disease severity and significantly increased survival rate (Fig. 3d). Taken together, these findings suggest that expression of *PsCRN115* improves plant resistance to two *Phytophthora* pathogens.

### Upregulated defence-related genes and promoted accumulation of H_2_O_2_ in *PsCRN115*-expressing *N. benthamiana*

**Figure 4:**
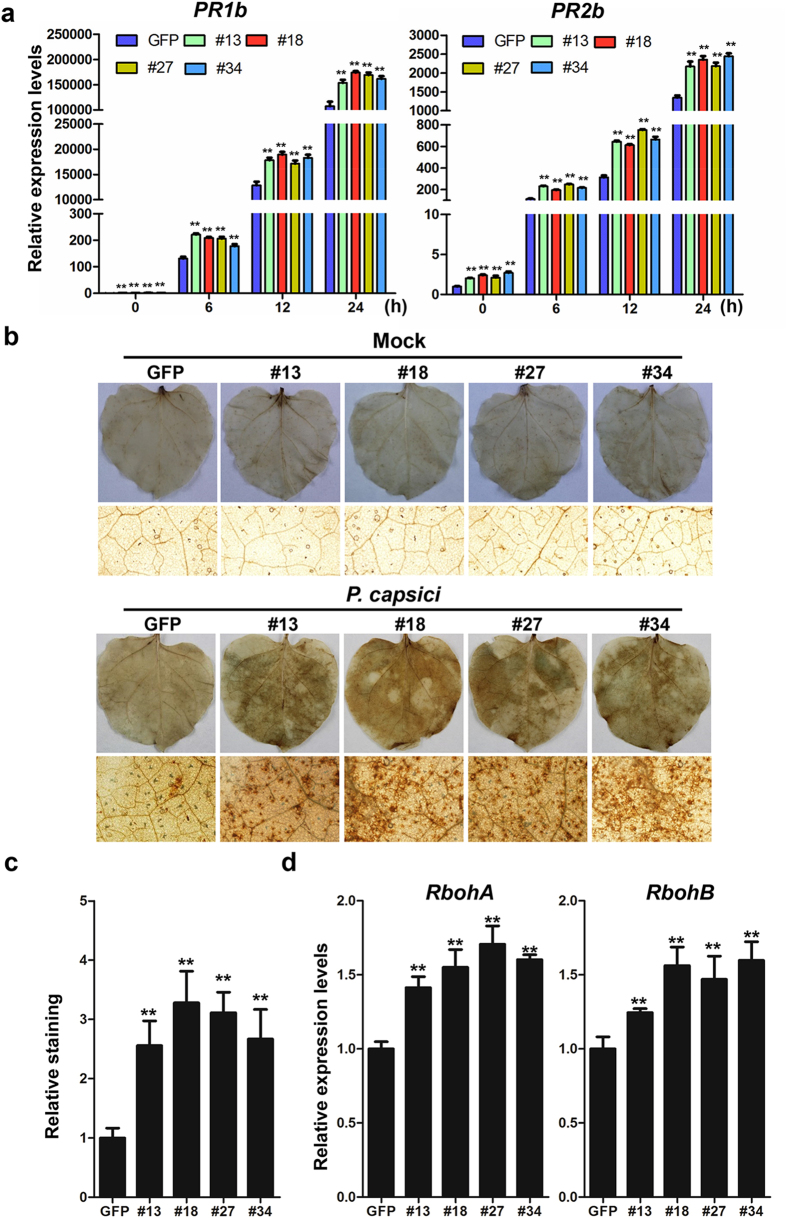
*PsCRN115* mediates upregulation of pathogenesis-related genes and H_2_O_2_ accumulation in *N. benthamiana*. (**a**) Expression of *PR1b* and *PR2b* genes. Samples were collected at the indicated time points upon infection with *P. capsici* zoospores. The relative expression levels were standardized to the *EF1α* gene. Error bars represent standard errors (**, P < 0.01, Dunnett’s test). (**b**) Increased H_2_O_2_ accumulation in *PsCRN115*-transgenic plants. H_2_O_2_ accumulation was visualized by DAB in transgenic *N. benthamiana* leaves at 12 h after inoculation with water (mock) or *P. capsici* zoospores. Detached leaves were stained with DAB solution as described in the Methods section. Microscopic observations of the DAB-stained leaves of *N. benthamiana* are shown at the bottom. (**c**) Relative intensity of DAB staining in *P. capsici*-infected *N. benthamiana*. Values are means ± SE of four biological replicates. Asterisks indicate significant differences between *GFP*- and *PsCRN115*-transgenic plants (**, P < 0.01, Dunnett’s test). (**d**) Expression levels of ROS-producing genes *RbohA* and *RbohB*. Asterisks indicate significant differences between *GFP*- and *PsCRN115*-transgenic plants (**, P < 0.01, Dunnett’s test).

To preliminarily explore the resistance to *Phytophthora* pathogens in transgenic plants, expression of defence-related genes were analysed using real-time qPCR. Since the salicylic acid (SA)-signalling pathway contributes positively to resistance against hemibiotrophic *Phytophthora* pathogens, expression levels of *PR1b* and *PR2b*, two marker genes in this pathway, were analysed. Expression levels of these defence-related genes were significantly higher in *PsCRN115*-transgenic *N. benthamiana* during *P. capsici* infection compared to in *GFP* lines (Fig. 4a). These results suggested that expression of *PsCRN115* might improve plant resistance to *Phytophthora* pathogens by upregulating the expression levels of defence-related genes.

To increase our understanding of the mechanism that leads to resistance to *Phytophthora* pathogens, we analysed the accumulation of H_2_O_2_ in plant leaves using the diamino-benzidine (DAB) staining method^38^. Very weak staining was observed in both *PsCRN115*- and *GFP*- transgenic plants under mock treatment with water, and no significant differences in staining strength were observed (Fig. 4b). By contrast, dark staining was observed in both *P. capsici-*infected *PsCRN115*- and *GFP*-transgenic plants (Fig. 4b). However, DAB staining was significantly stronger in *PsCRN115*-transgenic *N. benthamiana* compared to *GFP*-expressing plants challenged with *P. capsici* zoospores (Fig. 4b,c). To investigate the possible mechanisms underlying the elevated H_2_O_2_ accumulation in *PsCRN115*-transgenic plants, we detected the expression levels of genes encoding ROS-producing proteins; namely, respiratory burst oxidase homologues (*RbohA* and *RbohB*). As shown in Fig 4d, there was a minor but significant increase in expression of ROS-producing genes in *PsCRN115*-transgenic lines compared to *GFP*-expressing lines. This result suggested that the increased levels of H_2_O_2_ accumulation in *PsCRN115*-transgenic plants might be caused by upregulation of ROS-producing genes. Taken together, these results suggested that improved resistance to *Phytophthora* pathogens in *PsCRN115*-transgenic plants is likely caused by upregulation of defence-related genes and promotion of H_2_O_2_ accumulation.

### *PsCRN115* expression improves plant tolerance to salt stress

**Figure 5:**
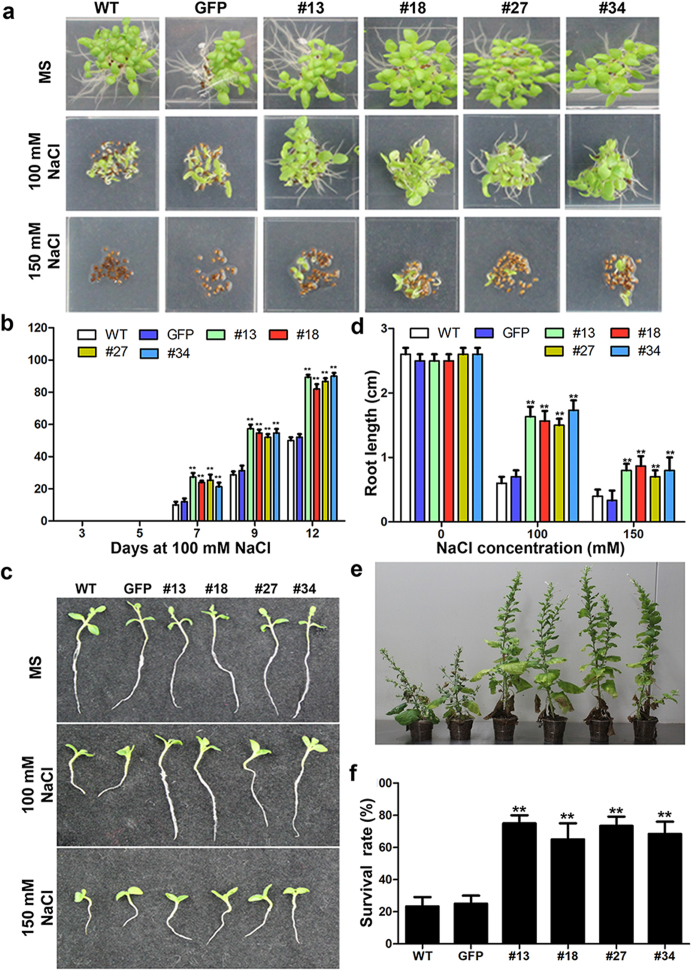
Increased salt tolerance in transgenic plants overexpressing *PsCRN115*. (**a**) Seed germination in the presence of specific NaCl concentrations 12 days after sowing. (**b**) Germination rates of the plants under normal and NaCl treatments. Germination rate was scored at the indicated days. Data represent the means ± SE of three independent experiments (**, P < 0.01, Dunnett’s test). (**c**) The post-germination seedling growth of plants. Seeds were germinated on MS medium for 4 d and transferred to MS medium supplemented with 100 or 150 mM NaCl. The photographs were taken 12 d after germination. (**d**) Root length of the seedlings 12 d after germination. Data represent the means ± SE of three independent measurements (**, P < 0.01, Dunnett’s test). (**e**) Effect of salt stress on plant growth in soil. Plants were treated with 150 mM NaCl for 2 weeks and photographed at 15 weeks after germination. (f) Survival rates of plants. Data represent the means ± SE of three independent measurements (**, P < 0.01, Dunnett’s test).

Biotic and abiotic stress responses converge in the stress signalling networks^39^, and PCD is also important for abiotic stresses^40^. We evaluated whether *PsCRN115* expression affected stress tolerance to salt and drought. No significant differences in seed germination were observed between *PsCRN115* and *GFP* lines on normal MS medium without NaCl (Fig. 5a and Supplementary Fig. S4). However, a significant increase in germination rates was observed in *PsCRN115* lines compared with WT and *GFP* lines in the presence of 100 mM NaCl (Fig. 5a,b). No seeds of WT and *GFP* lines germinated on the MS medium containing 150 mM NaCl (Fig. 5a and Supplementary Fig. S4); by contrast, about 10% of the seeds of the *PsCRN115* lines germinated 12 d after sowing (Fig. 5a and Supplementary Fig. S4). To confirm the increased tolerance to salt stress, the post-germination growth of the transgenic plants were tested. Seeds of the control and transgenic lines were allowed to germinate on normal MS plates for 4 d and then transferred onto MS medium containing different NaCl concentrations. The growth of seedlings was monitored by measuring their root lengths. The root lengths were significantly longer in *PsCRN115*-transgenic lines than control plants in the presence of 100 and 150 mM NaCl (Fig. 5c,d). Furthermore, we observed that the growth of the control plants (8-week-old) was considerable slower than the *PsCRN115*-transgenic lines 2 weeks after NaCl treatment (Fig. 5e). Nearly 80% of control plants died 3 weeks after treatment. In contrast, more than 70% of the *PsCRN115*-transgenic lines survived after treatment (Fig. 5f). These results indicated that expression of *PsCRN115* improved plant salt tolerance in *N. benthamiana*.

### *PsCRN115* expression enhances drought tolerance in plants

**Figure 6:**
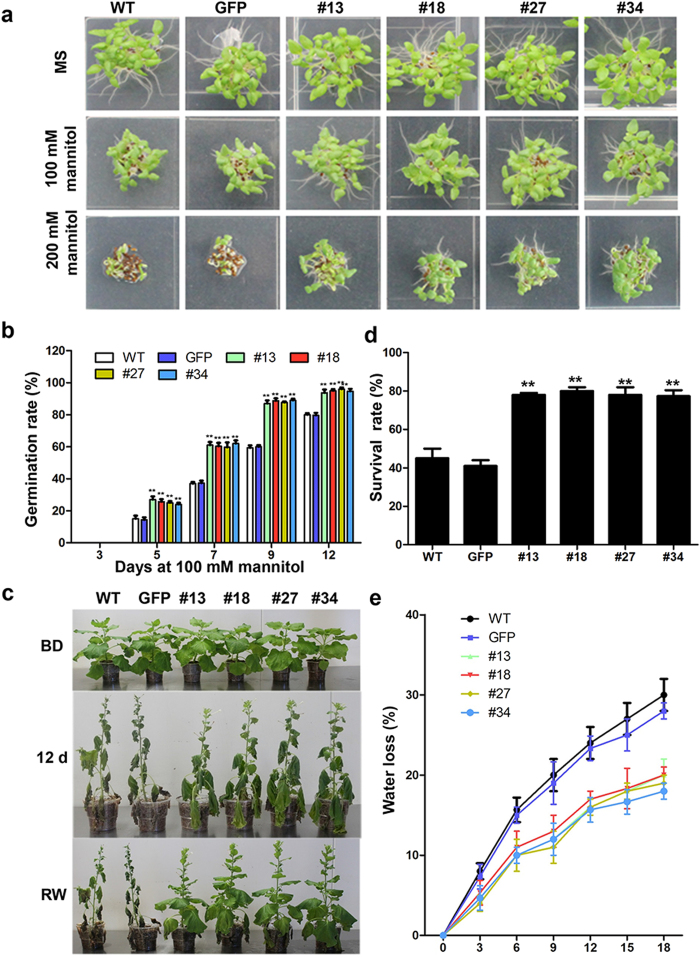
*PsCRN115* expression enhances drought tolerance in transgenic plants. (**a**) Seed germination at 12 days after sowing. (**b**) Germination rates. The data was scored and calculated at the indicated time points when treated with mannitol (**, P < 0.01, Dunnett’s test). (**c**) Phenotypes of plants submitted to drought stress at the vegetable stage. Water was withheld from 8-week-old plants for 12 d, after which the plants were watered for 2 d to allow them to recover. BD, before drought treatment; RW, re-watering. (**d**) Survival rates of plants under drought stress. Data represent the means ± SE of three independent experiments (**, P < 0.01, Dunnett’s test). (**e**) Water loss from the detached leaves of the indicated plants. The rate of water loss was calculated based on the loss of fresh weight in the samples. Bars indicate the standard error of 10 biological replicates.

To assess whether the expression of *PsCRN115* affects plant responses to other abiotic stresses, we sowed seeds of *PsCRN115* lines and control plants onto MS agar medium containing 100 or 200 mM mannitol, a widely used condition to mimic drought stress treatment^41^. The germination of control plant seeds was severely inhibited with increasing mannitol concentrations (Fig. 6a, Supplementary Fig. S5 online). However, the germination rate of *PsCRN115*-transgenic plants was significantly higher than control plants at 12 d after sowing on MS ager medium containing 100 or 200 mM mannitol (Fig. 6b and Supplementary Fig. S5). After 12 days of drought treatment of 8-week-old plants, WT and *GFP* lines were completely wilted, whereas the *PsCRN115*-transgenic plants were less affected (Fig. 6c). Two days after resumption of watering, the *PsCRN115*-transgenic plants recovered more rapidly than control plants (Fig. 6c). The survival rate of *PsCRN115*-transgenic plants was significantly higher than control plants after drought treatment (Fig. 6d). In addition, the rate of water loss from the detached leaves of *PsCRN115*-expressing plants was lower than that from detached leaves of WT and *GFP* plants under dehydration conditions (Fig. 6e). These results indicated that the *PsCRN115*-transgenic plants were more tolerant to drought stress.

### Genome-wide expression analysis in transgenic lines

To explore the mechanisms of biotic and abiotic stress tolerance underlying *PsCRN115*-expressing *N. benthamiana*, digital gene expression (DGE) tag profiling was performed to determine the differential gene expression between *PsCRN115*- and *GFP*- transgenic lines in leaves of 8-week-old plants under normal growth conditions. We observed 273 genes were significantly up- or down-regulated by over four-fold in *PsCRN115*-transgenic lines compared to *GFP* lines (Supplementary Table S1). This result suggested that *PsCRN115* had a significant impact on global gene expression profiles in *N. benthamiana*.

**Figure 7:**
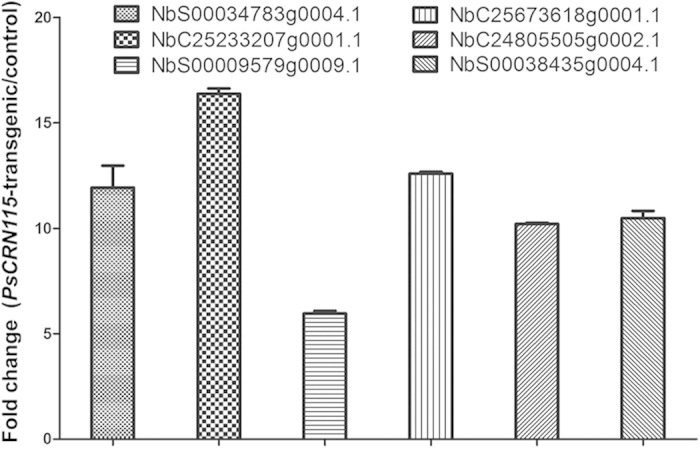
Validation of upregulated genes using quantitative RT-PCR. Relative gene expression between *PsCRN115*-transgenic plants and *GFP* lines was determined using qRT-PCR. *N. benthamiana EF1α* gene was used as a reference.

Among the upregulated genes, more than 10% belong to heat shock protein genes and cytochrome P450 genes (Table 1), which was not observed in downregulated genes (Supplementary Table S1). Eight HSPs were upregulated by at least six-fold in *PsCRN115*-transgenic plants compared to *GFP* lines (Table 1), suggesting that *PsCRN115-*trangenic plants acquire abiotic tolerance by accumulating more HSPs before stress conditions. Five cytochrome P450 genes were upregulated by at least four-fold in *PsCRN115*-expressing plants compared to *GFP* lines (Table 1), suggesting the P450 may play a role in plant stress resistance. Interestingly, a Bcl-2 binding anthanogene-1 gene, which functions in regulating apoptosis-like processes during pathogen attack and abiotic stress^42^, was upregulated by 5.31-fold in *PsCRN115*-trangenic plants compared to *GFP* lines (Supplementary Table S1). To evaluate the quality of the sequencing results, expression levels of 3 HSP genes (*NbS00034783g0004.1*, *NbC25233207g0001.1*, *NbS00009579g0009.1*), and 3 P450 genes (*NbC25673618g0001.1*, *NbC24805505g0002.1* and *NbS00038435g0004.1*) in Table 1 were determined using quantitative RT-PCR (Fig. 7). The RT-PCR analyses confirmed the results of DGE analysis.

Furthermore, we performed Blast2GO annotations to gain a general picture of the functions of genes identified by DGE approach. The upregulated unigenes are categorized into 20 functional groups in the three ontologies. The categories “cell part”, “organelle”, “intracellular” and “response to stress” are dominant (Supplementary Fig. S6). By contrast, The downregulated unigenes are categorized into 17 functional groups, and “cell part”, “ion binding” and “cellular metabolic process” are dominant (Supplementary Fig. S6). These results suggest that the increased stress tolerance in *PsCRN115*-transgenic *N. benthamiana* was achieved by modulation of plant stress responses and modification of cellular metabolism and functions.

## Discussion

Genome-wide catalogues of effectors are becoming available for many plant pathogens, and effectors have been used to assist in plant breeding^43^. In addition to applying effectors as molecular markers for marker-assisted selection, effectors have been exploited to accelerate *R* gene cloning and specificity profiling^43^. However, these strategies are dependent on knowledge of the interaction between *R* and *Avr* genes. Can we use effectors directly to improve plant resistance? In this study, we showed that expression of a CRN effector PsCRN115 significantly improved plant disease resistance and abiotic stress tolerance in *N. benthamiana*.

Recent study on *P. capsici* showed that non-host resistance (NHR) is correlated with HR elicited by a single RxLR effector^11^. *N. benthamiana* is not a host of *P. sojae* pathogen, and expression of a *P. sojae* effector may elicit NHR. However, effector-triggered immunity (ETI) tends to be associated with HR and constitutive activation of ETI may lead to fitness costs^44^. In this study, we did not observe obvious developmental changes in *PsCRN115*-expressing *N. benthamiana*, suggesting that improvement of plant defence by *PsCRN115* in *N. benthamiana* is likely achieved by other mechanisms and not due to perception by an unknown R protein.

CRNs are conserved in oomycete pathogens and share conserved domains^17^, therefore, they could be classified as pathogen-associated molecular patterns (PAMPs)^33^. PAMP-triggered immunity (PTI) is generally characterised by the induction of a reactive oxygen species (ROS) burst, callose deposition, and expression of defence-related genes without any signs of PCD^45^. In this study, we observed that ROS accumulation and expression of defence-related genes were upregulated in *PsCRN115*-transgenic lines compared to the control. These defence responses were similar to PTI. However, treatment with PAMPs leads to constitutive activation of defence responses, which usually causes severe growth reduction^46^. We found that expression of *PsCRN115* did not result in an obvious growth reduction while the defence-related genes were weakly induced in transgenic plants under normal growth conditions. Therefore, PsCRN115 may function as a weak “PAMP” to activate plant defence. However, constitutive expression of *PsCRN115* in *N. benthamiana* did not lead to visible callose deposition, which is a marker of PTI. These results further suggest that the enhanced stress tolerance in transgenic plants may be achieved by other unknown mechanisms.

DGE analyses showed that many HSPs and cytochrome P450 genes were upregulated in *PsCRN115*-transgenic plants. It has been shown HSPs and cytochrome P450 play a role in biotic and abiotic stress responses^47^,^48^,^49^. Heat shock proteins (HSPs) can assist in protein refolding under stress conditions^47^ and play a crucial role in protecting plants against stress by re-establishing normal protein conformation and cellular homeostasis. Timely expression of HSPs before severe heat conditions is vital for plants to acquire thermotolerance^48^. This result suggests that *PsCRN115* expression renders the plant more resilient to abiotic stress by upregulating many HSPs. Cytochrome P450s play crucial roles in the biosynthesis of a variety of endogenous lipophilic compounds, such as fatty acids, phytoalexins, brassinolides, and gibberellins. Expression of cytochrome P450 genes was induced by various biotic and abiotic stresses, indicating that they may be involved in the regulation of plant defence^49^. This result suggests that PsCRN115 improves plant disease resistance and abiotic stress tolerance by upregulating expression of HSPs and cytochrome P450.

The majority of CRNs are localised to plant nuclei^13^,^17^, and we found that PsCRN115 also targets the plant nucleus under biotic and abiotic stresses, indicating that CRNs may target and perturb host nuclear processes. However, it remains unclear whether PsCRN115 regulates expression of plant genes by directly binding to plant genomic DNA or indirectly by interfering with other proteins, such as histone proteins and transcriptional factors. Moderate levels of ROS may function as signalling molecules to promote cell survival, whereas severe increases in ROS can trigger cell death^50^. However, biotic and abiotic stresses usually lead to the overexpression of ROS, which are toxic and cause damage to plants^51^,^52^. We recently showed that PsCRN115 interacts with plant catalases, essential enzymes for ROS-scavenging. PsCRN115 may enhance the ability of plants to scavenge reactive oxygen species by stabilising catalases^36^. This may explain why PsCRN115 can improve plant tolerance to biotic and abiotic stresses.

PCD is involved in responses to biotic and abiotic stress stimuli^35^,^40^. We showed that PsCRN115 not only suppressed cell death triggered by some elicitors, but also suppressed PCD triggered by heat shock treatment. Interestingly, a Bcl-2 binding anthanogene-1 gene was upregulated in *PsCRN115*-transgenic lines based on DGE analyses. It was reported that this gene regulates apoptosis-like processes during pathogen attack and abiotic stress^42^. These results suggest that PsCRN115 improves biotic and abiotic stress tolerances by suppressing cell death triggered by these conditions.

In conclusion, we showed that expression of a CRN effector, *PsCRN115*, from *P. sojae* enhanced plant tolerance to biotic and abiotic stresses, suggesting that oomycete effectors can be used directly to improve plant defence against biotic and abiotic stresses.

## Methods

### Plant material and growth conditions

*Nicotiana benthamiana* seeds were surface sterilised and planted on Murashige and Skoog (MS) medium for germination under greenhouse conditions. Three-leaf stage *N. benthamiana* seedlings were transferred to soil and maintained under greenhouse conditions at 25 ± 1 °C with a 16 h light/8 h dark cycle.

### Vector construction and genetic transformation

The *PsCRN115* gene (lacking the predicted secretory signal peptide) was amplified using specific primers (Supplementary Table S2) and inserted into the binary vector pBinGFP2 via *Bam*H I and *Xba* I. The recombinant plasmid was introduced into *Agrobacterium tumefaciens* strain EHA105 for *N. benthamiana* transformation using the leaf disc method, as reported previously^53^. The transformants were screened for kanamycin resistance (100 mg/L) and further verified by PCR. Plants transformed with the pBinGFP2 vector were used as controls.

### Protein extraction and Western blot analyses

*N. benthamiana* leaf tissues were ground in liquid nitrogen and mixed with protein extraction buffer (50 mM HEPS, 150 mM KCl, 1 mM EDTA, 0.1% triton X-100, adjust pH to 7.5 with KOH) supplemented with 1 mM DTT and 1 × protease inhibitor mixture (Cocktail, Roche). Suspensions were mixed and centrifuged at 12000 × g for 15 min at 4 °C. Total proteins were separated on 12% SDS-polyacrylamide gels and transferred to Immobi-lon-P^SQ^ polyvinylidene difluoride membranes. The membranes were washed with PBST (PBS with 0.1% Tween 20) for 3 min and then blocked in 5% non-fat milk for 1 h. Mouse monoclonal anti-GFP or -HA antibody (Sigma-Aldrich) was added at a ratio of 1:5000 and incubated for 2 h, followed by three washes with PBST. The membranes were then incubated with goat anti-mouse IRDye 800CW (Odyssey, Li-Cor) at a ratio of 1:10000 at room temperature for 40 min with shaking. After three washes with PBST, the membranes were visualised using an Odyssey imaging system with excitation at 700 and 800 nm.

### Confocal microscopy

*N. benthamiana* leaves were cut into small squares and immersed into PBS buffer containing 5 μg/mL DAPI for staining of the nuclei for 5 min. Fluorescence was visualised with a Zeiss LSM 710 confocal laser-scanning microscope (CLSM). The excitation wavelength used for GFP was 488 nm and 405 nm for DAPI. Leaf tissues of *GFP*-transgenic lines were used as controls.

### Callose deposition assay

To induce callose deposition, 40 μM flg22 was infiltrated into 6-week-old *N. benthamiana* leaves. Leaf discs of the leaves were destained in 95% ethanol and then incubated at 60 °C until chlorophyll was removed. The cleared leaf discs were washed with 70% ethanol and then rinsed with distilled water. The leaf discs were then immersed in 0.1% aniline blue in 150 mM K_2_HPO_4_, pH 9.5, and incubated in the dark for 1 h. The stained leaf discs were rinsed with distilled water and mounted in 50% glycerol and examined under a UV epifluorescence microscope (Olympus BX71).

### Transient protein expression *in planta*

*A.tumefaciens* GV3101 was used to deliver T-DNA constructs into *N. benthamiana* leaves. Overnight *A.tumefaciens* cultures were harvested by centrifugation at 3500 × g for 5 min, washed with 10 mM MgCl_2_ three times, and resuspended in infiltration buffer (10 mM MgCl_2_, 10 mM MES, pH 5.6, and 150 μM acetosyringone) to an OD_600_ of 0.1 prior to infiltration into the entire leaf or leaf sections.

### Trypan blue staining

Cell death in *N. benthaminana* leaf tissues was detected using trypan blue staining. Trypan blue solution contains 10 g of phenol, 10 mL of lactic acid, 10 mL of glycerol, 10 mL of distilled water, and 20 mg of trypan blue (Sigma-Aldrich). After 12 h or 24 h recovery from heat shock treatment, leaf tissues were soaked in boiling trypan blue solution for 3 min and incubated for 5 h. Samples were then destained in chloral hydrate solution (250%, w/v) and photographed.

### *Phytophthora* infection assays

*Phytophthora parasitica* Pp025 and *Phytophthora capsici* Pc35 were routinely grown on 10% (v/v) V8 juice agar plates at 25 °C in the dark. Zoospores were prepared as reported previously^53^,^54^. For detached leaves, *Phytophthora* infection assays were performed using droplet inoculations of 10 μL of zoospore suspensions with a concentration of 50 zoospores/mL on the abaxial surface. The phenotype was monitored within 48 hours, and photographs were taken at 36 hours post-inoculation. For whole seedlings, the infection assays were performed by dipping roots into zoospore suspensions. The *GFP-*transgenic lines were used as controls. The inoculated plants were maintained in a moist chamber, and disease progression was monitored within 10 days. At least three independent experiments were performed for this assay. Dunnett’s test was used for statistical analysis (P < 0.01).

### RNA extraction and quantitative RT-PCR

Total RNA of *N. benthamiana* leaves was extracted using the RNAsimple Total RNA Kit (Tiangen, China) according to the manufacturer’s instructions. The cDNA was generated using the PrimeScript^™^ RT reagent Kit (TaKaRa). Real-time quantitative PCR was performed with SYBR Green fluorescence detection in a quantitative PCR thermal cycler (ABI PRISM 7300, Applied Biosystems). Each reaction was prepared using 2 μL of cDNA, 10 μL of SYBR^®^ Premix ExTaq (TaKaRa), 0.2 μM gene-specific primers (primer sequences in Table S2), and 0.4 μL of ROX reference dye in a total volume of 20 μL. The cycling conditions were as follows: 95 °C for 30 s, 40 cycles of 95 °C for 5 s and 60 °C for 31 s to calculate cycle threshold values, followed by a dissociation program of 95 °C for 15 s, 60 °C for 1 min, and 95 °C for 15 s to obtain melt curves. The *N. benthamiana EF1*α gene was used as an internal reference gene to calculate relative transcriptional levels.

### DAB staining

H_2_O_2_ was detected *in situ* using DAB staining^38^ with minor modifications. Briefly, *N. benthamiana* leaves were detached and inoculated with *P. parasitica* zoospores. The infected leaves 8 h after inoculation were soaked in DAB solution and maintained for 8 h at 25 °C. The leaf tissues were fixed in 95% ethanol and photographed.

### Salt and drought stress analyses

For salt treatment, T3-generation *PsCRN115*- and *GFP*-transgenic seeds were surfaced-sterilised and sown on MS agar medium supplemented with 0, 100, or 150 mM NaCl. The germination rate was measured daily after sowing. The root length was measured at 12 d after germination. For drought treatment, the seed germination rate was measured using the method described above. To evaluate drought tolerance during vegetative growth, water was withheld completely from 8-week-old *PsCRN115*- and *GFP*-transgenic lines for 12 d, after which the plants were watered for 2 d to allow them to recover. For water loss measurements, detached leaves of transgenic plants were incubated at 37 °C and weighed on an electronic balance at the indicated time points. The rate of water loss was plotted as the loss in leaf weight over time. Salt and drought stress analyses were repeated at least three times. Dunnett’s test was used for statistical analysis (P < 0.01).

### Digital gene expression profiling analysis

Total RNA was isolated from *PsCRN115*- and *GFP*- transgenic *N. benthamiana*, respectively, followed by Illumina sequencing using a HiSeq2000 to produce 100 bp paired-end data. All obtained clean reads were mapped to *N. benthamiana* reference gene sequences using Tophat^55^ with default parameters. The normalised gene expression level for each gene was calculated using the reads per kilo bases per million reads (RPKM) method^56^. The statistical significance of the differentially expressed genes was determined using GFOLD software (GFOLD > 1 or GFOLD < −1; log2 (fold change) >2 or log2 (fold change) < −2)^57^. For functional annotation, distinct sequences were BLAST against the NCBI NR database with an E-value cut-off of 10^−5^.

## Additional Information

**How to cite this article**: Zhang, M. *et al*. A *Phytophthora sojae* cytoplasmic effector mediates disease resistance and abiotic stress tolerance in *Nicotiana benthamiana*. *Sci. Rep*. **5**, 10837; doi: 10.1038/srep10837 (2015).

## Supplementary Material

Supplementary Information

## Figures and Tables

**Table 1:** List of significantly up-regulated heat shock protein and cytochrome P450 genes

Gene ID	Annotation	Fold change
NbS00034783g0004.1	Class II heat shock protein	18.12
NbC25233207g0001.1	Class I heat shock protein	14.95
NbS00040816g0003.1	Class I heat shock protein	10.70
NbS00025860g0007.1	Class I heat shock protein	7.89
NbS00007018g0006.1	Class II heat shock protein	7.01
NbS00004735g0001.1	Class I heat shock protein	6.39
NbS00034783g0009.1	Class II heat shock protein	6.16
NbS00009579g0009.1	Class I heat shock protein	6.06
NbC25673618g0001.1	Cytochrome P450	16.55
NbC24805505g0002.1	Cytochrome P450	12.74
NbS00038435g0004.1	Cytochrome P450	7.78
NbS00010554g0005.1	Cytochrome P450	7.75
NbS00018253g0004.1	Cytochrome P450	4.35
