# Correction: A *Phytophthora sojae* cytoplasmic effector mediates disease resistance and abiotic stress tolerance in *Nicotiana benthamiana*

**DOI:** 10.1038/s41598-026-67578-8

**Published:** 2026-09-11

**Authors:** Meixiang Zhang, Nasir Ahmed Rajput, Danyu Shen, Peng Sun, Wentao Zeng, Tingli Liu, Joseph Juma Mafurah, Daolong Dou

**Affiliations:** 1 Department of Plant Pathology, Nanjing Agricultural University, Nanjing, China; 2 Department of Plant Pathology, Sindh Agriculture University, Tandojam, Pakistan

Correction to: *Scientific Reports* https://doi.org/10.1038/srep10837, published online 03 June 2015

This Article contains errors in Figures 2c and 4b as a result of incorrect figure assembly.

In Figure 2c, the leaf images in the lower right panel for transgenic line #13 at 0 h and 24 h time points are incorrect. The images were inadvertently taken from the GFP control line under RT conditions. In addition, the time-point label printed inside this lower figure panel is incorrect. The position labelled 0 h corresponds to samples collected at 12 h post-treatment.

In Figure 4b, the microscopic image for transgenic line #34 in the P. capsici inoculation group is incorrect. The image was inadvertently taken from line #18.

The correct Figure 2c and Figure 4b and their accompanying legends are shown below.

**Fig. 2:**
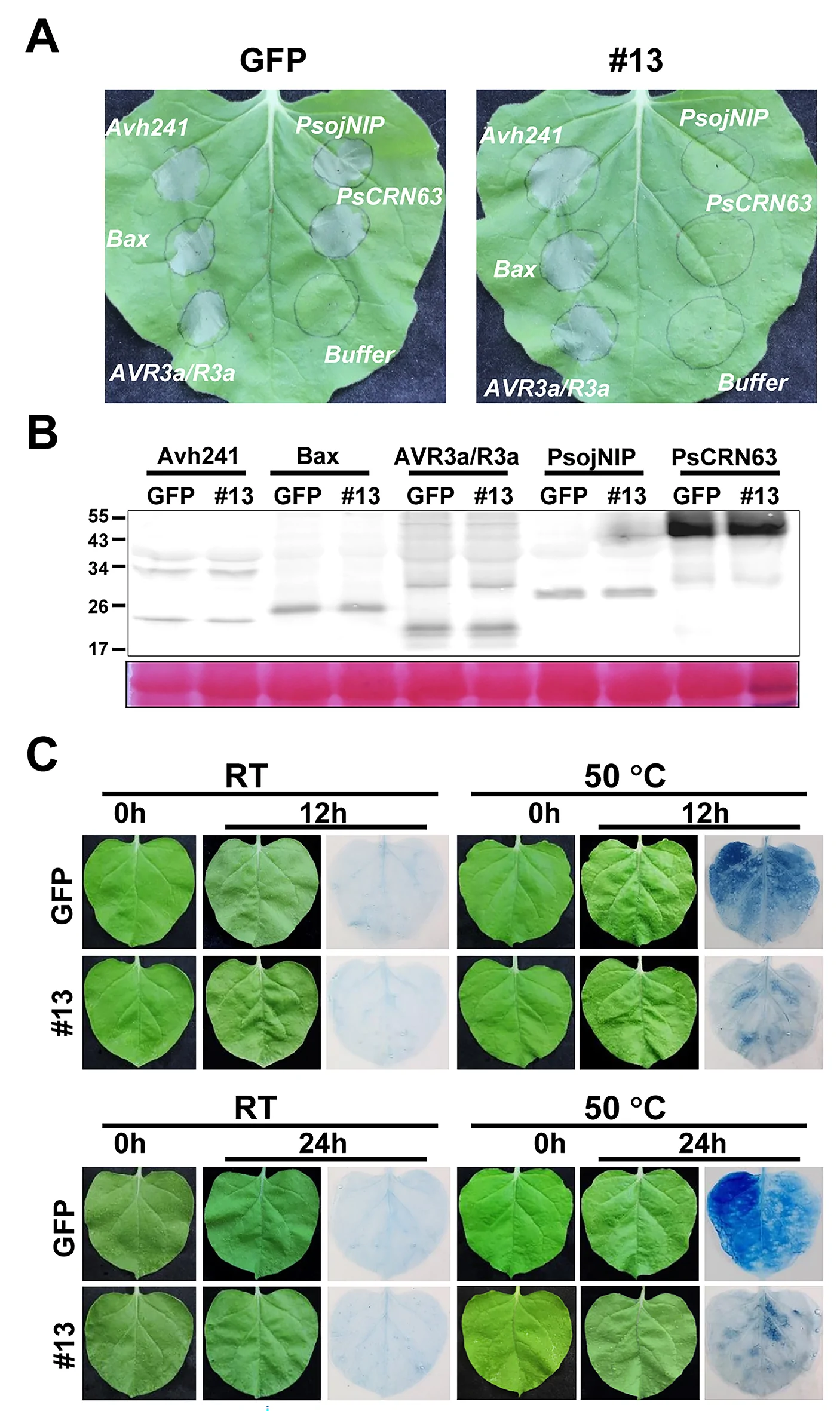
**Suppression of cell death in** ***PsCRN115*-expressing plants**. (**a**) Suppression of elicitor-triggered cell death in *PsCRN115*-transgenic *N. benthamiana*. The leaves were infiltrated with *Agrobacterium* GV3101 cells containing a PVX vector carrying *PsAvh241*, *Bax*, *Avr3a*/*R3a*, *PsojNIP* or *PsCRN63*. The photographs were taken 5 d after infiltration. (**b**) Confirmation of protein expression of the cell-death elicitors using Western blot analysis. Mouse monoclonal antibodies against the HA-epitope tag were used to detect expression of the cell-death elicitors fused to HA tags. (**c**) Heat-induced programmed cell death in transgenic *N. benthamiana*. Leaves of *GFP*- and *PsCRN115*-transgenic plants were detached and floated on water at room temperature or 50 °C for 40 min in the dark. The photographs were taken at 0, 12 and 24 h after treatment. The treated leaves were stained with trypan blue to visualise cell death. The results were reproducible in each transgenic line in at least three replicates and #13 was shown as an example.

**Fig. 4:**
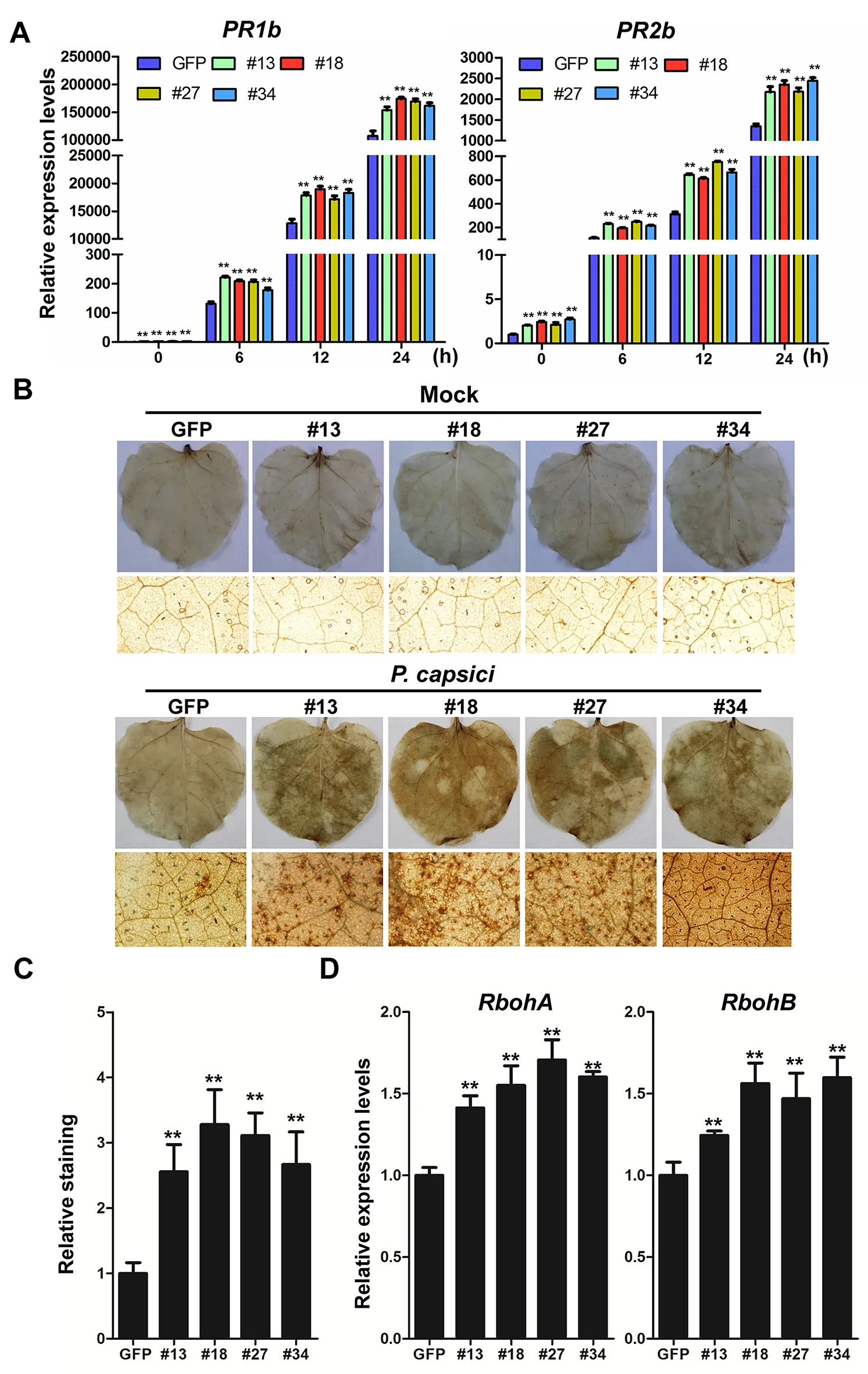
***PsCRN115*** **mediates upregulation of pathogenesis-related genes and H**_**2**_**O**_**2**_ **accumulation in** ***N. benthamiana***. (**a**) Expression of *PR1b* and *PR2b* genes. Samples were collected at the indicated time points upon infection with *P. capsici* zoospores. The relative expression levels were standardized to the *EF1α* gene. Error bars represent standard errors (**, P < 0.01, Dunnett’s test). **(b)** Increased H_2_O_2_ accumulation in *PsCRN115*-transgenic plants. H_2_O_2_ accumulation was visualized by DAB in transgenic *N. benthamiana* leaves at 12 h after inoculation with water (mock) or *P. capsici* zoospores. Detached leaves were stained with DAB solution as described in the Methods section. Microscopic observations of the DAB-stained leaves of *N. benthamiana* are shown at the bottom. **(c)** Relative intensity of DAB staining in *P. capsici*-infected *N. benthamiana*. Values are means ± SE of four biological replicates. Asterisks indicate significant differences between *GFP*- and *PsCRN115*-transgenic plants (**, P < 0.01, Dunnett’s test). **(d)** Expression levels of ROS-producing genes *RbohA* and *RbohB*. Asterisks indicate significant differences between *GFP*- and *PsCRN115*-transgenic plants (**, P < 0.01, Dunnett’s test).

