# Supplementary material for: A Phytophthora sojae cytoplasmic effector mediates disease resistance and abiotic stress tolerance in Nicotiana benthamiana
Source: Sci Rep. 2015 Jun 3;5:10837. doi: 10.1038/srep10837 (PMC4454142; doi:10.1038/srep10837)
Supplement: Supplementary Information [file srep10837-s1.pdf]

A *Phytophthora sojae* cytoplasmic effector mediates disease resistance and abiotic stress tolerance in *Nicotiana benthamiana*

Meixiang Zhang[†], Nasir Ahmed Rajput[†], Danyu Shen, Peng Sun, Wentao Zeng, Tingli Liu, Joseph Juma Mafurah, Daolong Dou*

Department of Plant Pathology, Nanjing Agricultural University, Nanjing, China

Additional title page Footnotes

[†], M.Z. and N.A.R. contributed equally to this work.

*To whom all correspondence should be sent:

Name: **Daolong Dou**

Address: #1 Weigang Rd, Nanjing Agricultural University, Nanjing, China

Telephone number: +86-025-84396973

e-mail address: ddou@njau.edu.cn

Supplementary information

Supplementary figures

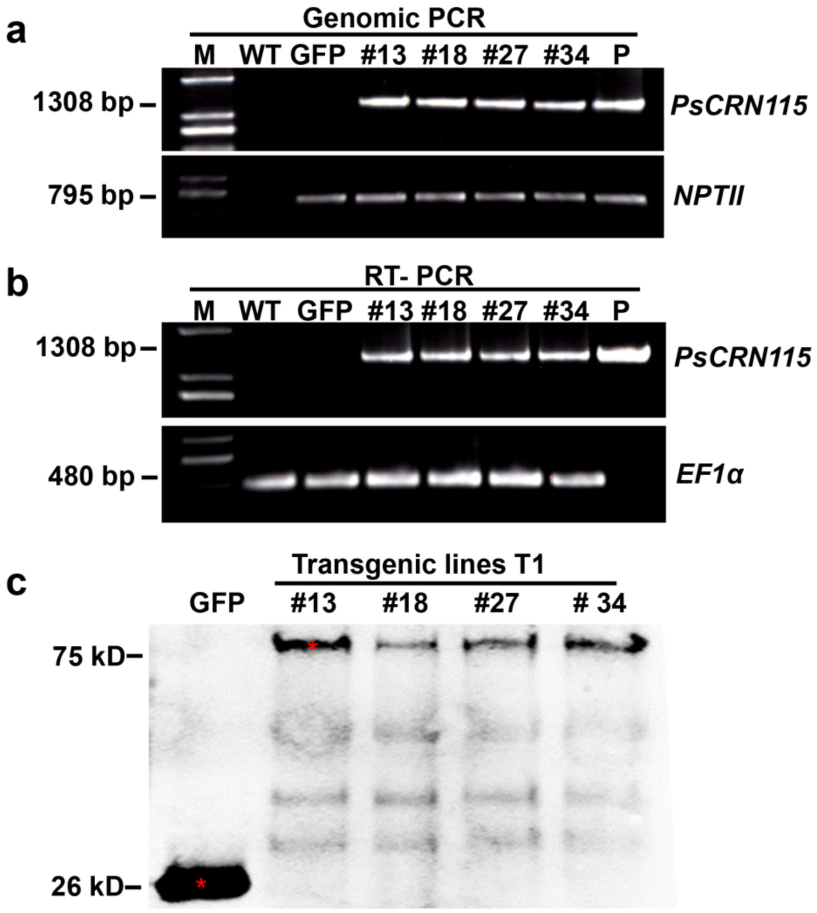

Figure S1. Generation of the *PsCRN115*-transgenic *N. benthamiana*. (a) Confirmation of transgene integration by genomic PCR. (b) Detection of *PsCRN115* expression in transgenic plants using RT-PCR. M, DNA marker; WT, wild-type *N. benthamiana*; GFP, *GFP*-transgenic *N. benthamiana*; #13, #18, #27 and #34, four independent *PsCRN115*-transgenic lines; P, the plasmid used for transformation as a positive control. *EF1α*, *N. benthamiana* internal reference gene *NbEF1α*. (c) Detection of GFP: PsCRN115 protein expression using Western blot. The expected target bands were indicated by asterisks.

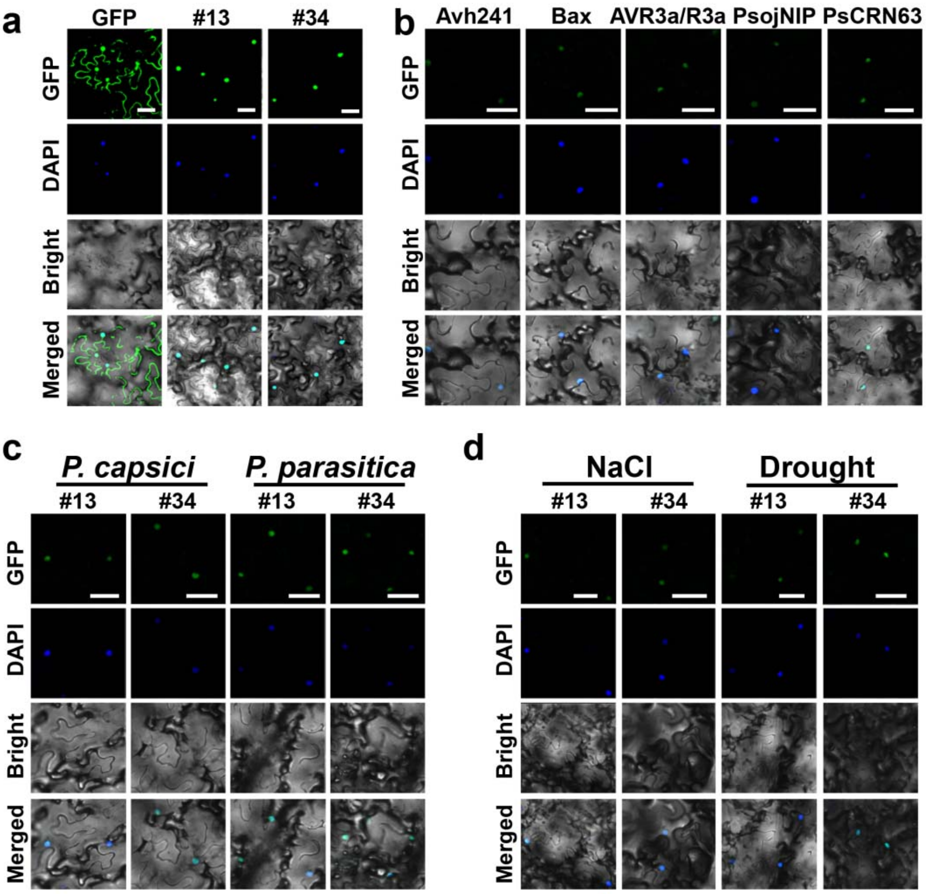

Figure S2. Subcellular localization of GFP:PsCRN115 fusion protein in transgenic *N. benthamiana* under normal and stress conditions. The pictures were taken using a confocal microscope. DAPI staining was used to show the nuclei. Scale bar represents 50 μm.

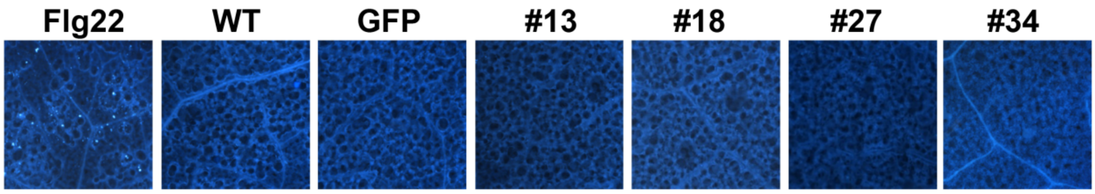

Figure S3. Detection of callose deposition in *PsCRN115*-transgenic *N .benthamiana*. Leaves treated with flg22 (40 μM) was used as positive controls. Callose deposits were detected by aniline blue staining, and visualised under a UV epifluorescence microscope (Olympus BX71).

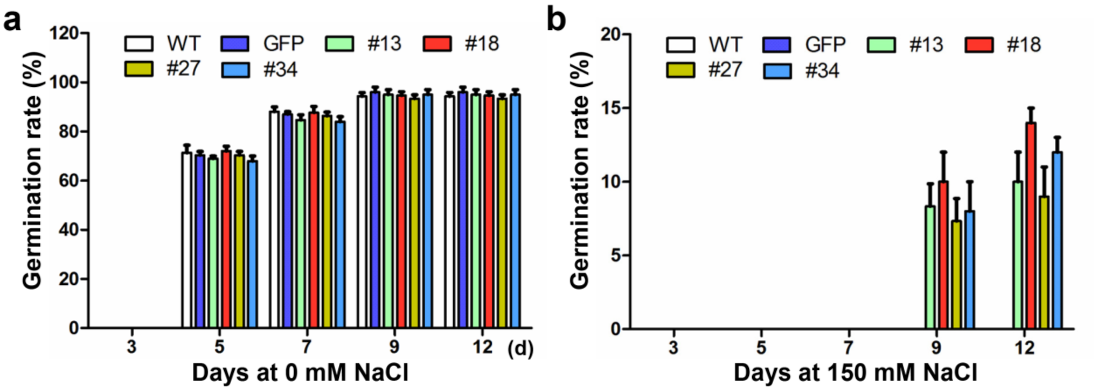

Figure S4. Germination rates of plants under salt stress. (a) Seed germination on MS medium containing 0 mM NaCl. (b) Seed germination on MS medium containing 150 mM NaCl. The results were based on daily scoring. The presented data are means ± SE of three independent experiments.

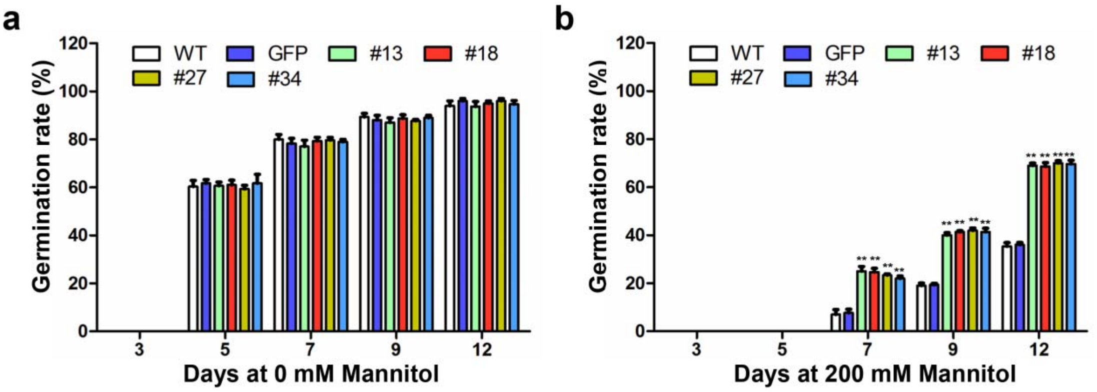

Figure S5. Germination rates of plants under mannitol treatment. (a) Seed germination on MS medium containing 0 mM mannitol. (b) Seed germination on MS medium containing 200 mM mannitol. The results were based on daily scoring. The presented data are means ± SE of three independent experiments. Statistical significance was determined by Dunnett's test (**, P<0.01).

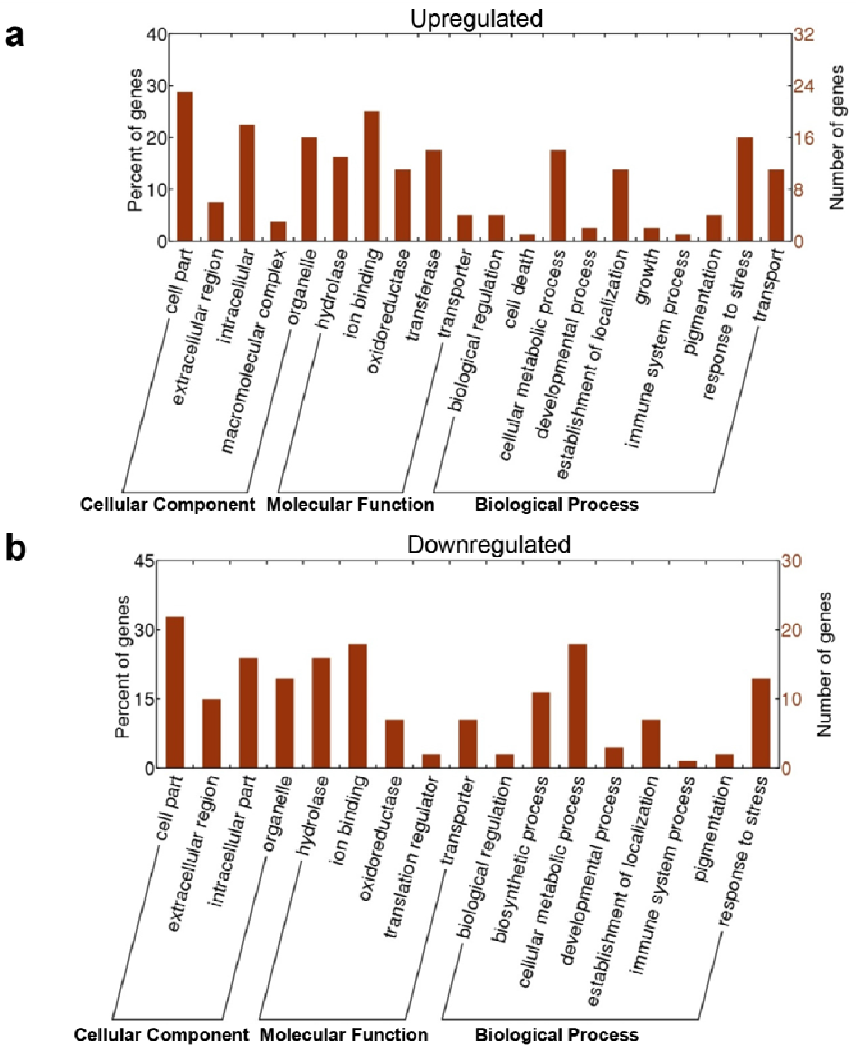

Figure S6. GO categories of the differentially expressed genes.

Supplementary tables

Table S1 Differentially expressed genes in *PsCRN115*-transgenic *N. benthamiana* compared to *GFP* lines.

Gene ID	GFP RPKM	PsCRN115 RPKM	Fold Change PsCRN115/ GFP	GFLOD value	Annotation	Validated by qPCR
NbS00040499g0025.1	0.13	0.84	4.01	-1.02192	Myosin XI F	
NbS00004242g0002.1	2.06	8.57	4.02	-1.34192	Aspartic proteinase nepenthesin 1	
NbS00001592g0014.1	0.32	1.60	4.03	-1.09157	Alkaline alpha galactosidase seed imbibition protein	
NbS00003883g0012.1	7.12	29.11	4.05	-1.56687	2 oxoglutarate/FeII dependent dioxygenase	
NbS00009622g0029.1	4.17	17.21	4.05	-1.47845	Auxin induced protein PCNT115	
NbS00001880g0007.1	1.18	5.09	4.07	-1.20612	Plastid lipid associated protein PAP/fibrillin	
NbS00016847g0019.1	1.29	5.58	4.07	-1.13822	Outer membrane lipoprotein blc	
NbS00019641g0010.1	2.67	11.22	4.09	-1.42264	Hydroxyproline rich glycoprotein	
NbS00054960g0005.1	0.99	4.37	4.10	-1.08551	UDP glucose salicylic acid glucosyltransferase	
NbS00012114g0012.1	0.66	3.02	4.10	-1.2285	Cellulose synthase C1 2 glycosyltransferase family 2 protein	
NbS00047765g0005.1	1.06	4.66	4.10	-1.39668	Serine/threonine protein kinase	
NbS00044318g0004.1	131.36	541.42	4.12	-1.80256	Proline rich protein	
NbS00016396g0001.1	77.30	319.63	4.13	-1.75701	Basic form of pathogenesis related protein 1	
NbS00025703g0006.1	5.45	23.36	4.22	-1.60571	Xyloglucan endotransglucosylase	
NbS00011039g0009.1	0.75	3.51	4.24	-1.20692	RNA polymerase sigma 70 factor	
NbS00009031g0001.1	4.21	18.42	4.29	-1.33758	Uncharacterized protein	
NbS00006819g0015.1	0.93	4.35	4.32	-1.23695	Trigger factor protein	
NbS00028015g0007.1	0.58	2.87	4.34	-1.39856	Pentatricopeptide repeat containing protein chloroplastic	

NbS00018253g0004.1	3.17	14.09	4.35	-1.57096	Cytochrome P450	
NbS00012812g0001.1	23.29	101.66	4.35	-1.77301	Pathogenesis related protein STH 2	
NbS00019467g0006.1	1.41	6.46	4.35	-1.27689	Heavy metal associated domain containing protein expressed	
NbS00029493g0011.1	2.70	12.19	4.38	-1.18145	Basic 7S globulin 2	
NbS00000009g0012.1	1.25	5.83	4.39	-1.64879	ABC transporter G family member 36	
NbS00022963g0016.1	0.68	3.34	4.41	-1.28236	Lysosomal Pro X carboxypeptidase	
NbS00029380g0012.1	2.60	11.89	4.44	-1.68135	Protein CHUP1 chloroplastic	
NbS00016427g0019.1	0.35	1.94	4.51	-1.72384	Unknown protein	
NbS00024265g0004.1	4.51	20.79	4.53	-1.70263	Pectinacetylesterase protein Fragment	
NbS00000680g0003.1	0.99	4.89	4.56	-1.46499	Sulfate transporter	
NbS00028378g0002.1	10.77	49.82	4.59	-1.84184	BURP domain containing protein	
NbS00014862g0017.1	1.80	8.66	4.61	-1.77896	Tetratricopeptide repeat TPR	
NbS00001173g0014.1	1.12	5.65	4.70	-1.39539	Auxin efflux carrier	
NbS00018169g0009.1	0.94	4.77	4.70	-1.39377	Serine carboxypeptidase	
NbS00008946g0009.1	1.34	6.70	4.73	-1.34197		
NbS00013555g0004.1	4.18	20.68	4.85	-1.58857	Xyloglucan endotransglucosylase/hydrolase 9	
NbS00017657g0002.1	1.89	9.62	4.89	-1.61666	UDP glucosyltransferase family 1 protein	
NbS00030404g0006.1	0.68	3.72	4.91	-1.02663	P loop containing nucleoside triphosphate hydrolases	
NbS00030446g0004.1	0.61	3.42	4.93	-1.52796	Subtilisin protease	
NbS00001344g0014.1	0.03	0.57	5.10	-1.18972	Os01g0917200 protein Fragment	
NbS00050570g0003.1	1.45	7.82	5.12	-1.35329		
NbS00020801g0017.1	2.55	13.54	5.14	-1.61663	Epoxide hydrolase 2	
NbS00008008g0014.1	0.46	2.82	5.20	-1.31574	Nitrate transporter	
NbS00003547g0001.1	0.37	2.38	5.31	-1.00053	Bcl-2 binding anthanogene 1	
NbS00024269g0011.1	0.45	2.84	5.33	-1.58291	Pentatricopeptide repeat containing protein chloroplastic	
NbS00007957g0021.1	0.60	3.71	5.42	-1.71857		
NbS00027349g0010.1	0.57	3.52	5.43	-1.63904	J domain protein required for chloroplast accumulation response 1	

NbS00031400g0005.1	0.54	3.36	5.44	-1.02192	Alpha/beta Hydrolases	
NbS00018866g0003.1	2.38	13.41	5.46	-1.73185	Aldo/keto reductase	
NbS00044308g0002.1	0.71	4.33	5.48	-1.31574	UDP glycosyltransferase 73C1	
NbS00013338g0011.1	0.53	3.39	5.53	-1.05136	Violaxanthin de epoxidase chloroplastic	
NbS00058503g0006.1	0.71	4.39	5.54	-1.6592	Cellulose synthase	
NbS00007667g0008.1	0.15	1.30	5.55	-1.08561	Protein of unknown function DUF547	
NbS00015926g0011.1	0.67	4.23	5.64	-1.25231	UDP glycosyltransferase 73C3	
NbS00013500g0008.1	1.31	7.89	5.67	-1.29375	Unknown protein	
NbS00043176g0004.1	0.04	0.69	5.72	-1.18972	Armadillo/beta catenin repeat	
NbS00032996g0005.1	0.20	1.61	5.74	-1.15604	Rop guanine nucleotide exchange factor 1	
NbS00017189g0001.1	25.35	146.72	5.77	-2.03077		
NbS00045061g0011.1	0.33	2.40	5.78	-1.24231	Long chain alcohol oxidase FAO1	
NbS00004241g0002.1	2.83	16.94	5.81	-1.90862	Aspartic proteinase nepenthesin 1	
NbS00059116g0016.1	24.42	143.55	5.86	-2.23267	Chitinase	
NbS00023863g0103.1	3.01	18.18	5.88	-1.65078		
NbS00013984g0021.1	91.98	543.71	5.91	-2.35969	Chitinase	
NbS00054535g0001.1	0.52	3.60	5.98	-1.016	Chloroplastic group IIA intron splicing facilitator CRS1 chloroplastic	
NbS00009579g0009.1	0.72	4.89	6.06	-1.06491	Class I heat shock protein	Yes
NbS00011583g0011.1	0.05	0.79	6.07	-1.10474	Mate efflux	
NbS00009083g0002.1	0.79	5.31	6.09	-1.95925	Beta galactosidase	
NbS00034783g0009.1	0.59	4.17	6.16	-1.12657	Class II heat shock protein	
NbS00007957g0022.1	0.35	2.69	6.20	-1.34116		
NbS00030877g0008.1	0.89	6.09	6.23	-1.95197	AE family transporter anion exchange	
NbS00002197g0002.1	6.02	38.03	6.23	-2.0204	Glutathione S transferase parC	
NbS00004735g0001.1	1.66	11.15	6.39	-1.46103	Class I heat shock protein	
NbS00005291g0008.1	0.09	1.11	6.47	-1.71631	Sucrose phosphate synthase	
NbS00012539g0001.1	0.06	0.92	6.49	-1.01453	Serine/threonine protein phosphatase 2A regulatory subunit delta 1 isoform	
NbS00029951g0016.1	1.16	8.11	6.54	-2.14571	Unknown protein	
NbS00022964g0001.1	0.60	4.46	6.55	-1.50737	UDP glucosyltransferase 1	
NbS00022499g0003.1	0.27	2.40	6.72	-1.39367	Protein WAX2	

NbS00013984g0030.1	31.96	218.16	6.81	-2.41788		
NbS00059116g0008.1	3.54	24.83	6.85	-1.62462		
NbS00025192g0008.1	0.07	1.11	6.97	-1.50187	Tetratricopeptide repeat protein 7A	
NbS00006672g0042.1	0.05	0.98	6.97	-1.18972	Leucine rich repeat receptor protein kinase	
NbS00007018g0006.1	0.55	4.45	7.01	-1.35007	Class II heat shock protein	
NbS00011805g0001.1	1.61	12.16	7.19	-1.2426	CP12	
NbS00013343g0015.1	172.28	1254.38	7.28	-2.65588	Osmotin protein Fragment	
NbS00044431g0007.1	1.19	9.31	7.30	-1.69746	Peroxidase	
NbC26100965g0001.1	0.07	1.12	7.37	-1.10474	CBL interacting protein kinase 6	
NbS00033938g0008.1	1.88	14.59	7.43	-1.90394	Blue copper protein Fragment	
NbS00007957g0008.1	0.23	2.40	7.49	-1.62398		
NbS00003673g0010.1	88.80	673.07	7.57	-2.65843	Proteinase inhibitor I	
NbS00044570g0003.1	0.33	3.20	7.63	-1.52609	Transmembrane protein 205	
NbS00007307g0011.1	0.18	2.04	7.65	-1.05198	Ethylene responsive transcription factor 2a	
NbS00010554g0005.1	0.76	6.60	7.75	-2.13341	Cytochrome P450	
NbS00038435g0004.1	3.30	26.38	7.78	-2.45691	Cytochrome P450	Yes
NbS00025860g0007.1	1.60	13.34	7.89	-2.23463	Class I heat shock protein	
NbS00040444g0001.1	218.41	1727.60	7.91	-2.75922	Bifunctional inhibitor/lipid transfer protein/seed storage 2S albumin	
NbS00015751g0019.1	0.00	0.70	8.02	-1.67653	CHY zinc finger containing protein	
NbS00026613g0002.1	279.52	2259.85	8.08	-2.82498	Osmotin protein Fragment	
NbS00040444g0002.1	11.04	90.19	8.10	-2.4383		
NbS00040444g0003.1	16.66	138.80	8.29	-2.39912		
NbS00013631g0006.1	8.81	75.10	8.44	-2.74815	Purple acid phosphatase	
NbC25315011g0002.1	1.56	14.04	8.51	-1.17076	Peroxidase 4	
NbS00012817g0004.1	0.17	2.31	8.92	-1.61985	Serine/threonine protein kinase	
NbS00000956g0001.1	32.11	290.52	9.02	-2.81913		
NbS00030269g0107.1	2.46	23.32	9.15	-2.69126		
NbS00032813g0006.1	0.33	4.11	9.86	-2.02226	Peptide transporter	
NbS00040816g0003.1	0.32	4.41	10.70	-1.34895	Heat shock protein	
NbS00009619g0006.1	1.44	16.84	11.01	-2.70797	Heat stress transcription factor	
NbS00010925g0003.1	7.17	80.01	11.02	-2.86213	Uncharacterized protein LOC100782725	
NbS00057756g0005.1	0.39	6.90	14.27	-2.66446	Beta glucosidase	

NbC24295021g0001.1	0.56	9.57	14.61	-1.68359	Protein CHUP1 chloroplastic	
NbC25233207g0001.1	1.23	19.74	14.95	-1.59557	Class I heat shock protein	Yes
NbS00018954g0001.1	0.00	1.66	17.62	-1.67653	Terpene synthase 21	
NbS00034783g0004.1	0.15	4.48	18.12	-1.94559	Class II heat shock protein	Yes
NbS00007436g0026.1	0.39	10.02	20.56	-2.9996		
NbS00009355g0029.1	0.00	5.87	59.66	-1.27003		
NbS00002437g0006.1	1.43	6.64	4.42	-1.63673	Hypothetical protein VITISV_033286	
NbS00036752g0007.1	0.31	1.76	4.54	-1.29894	Receptor kinase RLK	
NbS00019972g0004.1	2.01	10.87	5.19	-1.90916	Alpha 1 4 glucan phosphorylase L 2 isozyme 2C chloroplastic/amyloplastic	
NbS00003844g0006.1	0.43	3.21	6.23	-1.21445	FAD binding domain containing protein	
NbS00006399g0017.1	0.28	2.47	6.79	-1.56236	Filament plant protein Fragment	
NbS00035831g0003.1	0.08	1.55	9.28	-1.34615	GNOM 1	
NbC24805505g0002.1	1.03	14.31	12.74	-2.08619	Cytochrome P450	Yes
NbC25673618g0001.1	0.22	5.17	16.55	-2.18426	Cytochrome P450	Yes
NbS00058627g0001.1	5.96	0.00	-60.61	1.87842	Acetyl coenzyme A cis 3 hexen 1 ol acetyl transferase	
NbS00040042g0003.1	4.58	0.00	-46.79	1.87842		
NbS00003530g0014.1	14.25	0.21	-46.18	3.42424	Heavy metal transport/detoxification	
NbS00008926g0005.1	6.65	0.24	-19.73	2.63452	Vacuolar sorting protein 4b	
NbS00027213g0001.1	18.83	0.86	-19.67	2.56937	Non specific lipid transfer protein 2	
NbS00042659g0003.1	10.93	0.52	-17.87	2.23609		
NbS00045440g0005.1	3673.5 6	220.34	-16.67	4.09306	Pathogenesis related protein R major form	
NbS00060234g0001.1	4.43	0.17	-16.49	1.94423	Phosphoglycerate mutase	
NbS00007655g0001.1	46.23	2.95	-15.18	2.96717	Non specific lipid transfer protein 2	
NbS00009733g0019.1	12.21	0.71	-15.18	2.39055	Major allergen Pru ar 1	
NbS00004868g0007.1	154.76	10.19	-15.06	3.41136	NBS LRR class disease resistance protein	
NbS00052547g0003.1	24.47	1.54	-14.95	3.19218	Nudix hydrolase 2	
NbS00004740g0003.1	14.48	1.04	-12.78	2.31342	Heavy metal associated isoprenylated plant protein 26	
NbS00049765g0001.1	23.68	1.76	-12.77	2.34656	Calcium binding protein CML45	
NbS00017574g0006.1	12.61	0.92	-12.48	2.92997	Cathepsin B cysteine proteinase	

NbS00004740g0002.1	3.73	0.21	-12.45	1.40554	Protein of unknown function DUF538	
NbS00027996g0004.1	36.45	2.94	-12.01	2.94605	Cell number regulator 1	
NbS00011121g0004.1	6.90	0.52	-11.25	1.9889	Receptor protein kinase At5g59670	
NbS00014933g0008.1	2.19	0.10	-11.24	1.66105		
NbS00025385g0006.1	39.41	3.42	-11.21	3.18331	Phospholipase A1 IIgamma	
NbS00020486g0001.1	35.70	3.11	-11.15	2.71265		
NbS00042659g0005.1	4532.80	406.80	-11.14	3.5387	PAR 1c protein	
NbS00028666g0002.1	3.76	0.25	-10.90	1.09596	WRKY transcription factor 16	
NbS00023311g0013.1	11.20	1.00	-10.29	2.85808	Solute carrier family 2 facilitated glucose transporter member 5	
NbS00025657g0014.1	2.70	0.17	-10.22	2.04628	Exostosin	
NbS00053606g0003.1	29.36	2.78	-10.22	3.05125	AAA ATPase	
NbS00032250g0015.1	1.46	0.06	-10.04	2.0072	Protein phosphatase 2C	
NbS00004308g0002.1	281.51	28.85	-9.73	3.13107		
NbS00003161g0001.1	2.23	0.14	-9.68	1.20656	Unknown protein	
NbS00012876g0003.1	3.00	0.22	-9.57	1.81955	Metacaspase	
NbC24762244g0001.1	2.42	0.16	-9.56	1.09596	Laccase	
NbS00006288g0006.1	2.40	0.16	-9.54	1.09596	Calcium binding protein CML10	
NbS00010244g0014.1	13.05	1.32	-9.24	2.71417	Inorganic phosphate transporter	
NbS00007314g0014.1	1.44	0.07	-9.17	1.66105	Eukaryotic initiation factor 4A 8	
NbS00005040g0005.1	2.92	0.23	-9.14	1.44299		
NbS00005579g0014.1	1.63	0.09	-9.08	1.40554	Exostosin	
NbS00003110g0006.1	2.89	0.23	-8.94	1.69141	Cathepsin B cysteine proteinase	
NbS00001820g0004.1	22.97	2.56	-8.68	2.57563		
NbS00008166g0001.1	8.22	0.86	-8.63	2.21688	Pto Serine/threonine kinase protein%2C resistance protein	
NbS00034797g0008.1	1.27	0.06	-8.57	2.23609	Calcium transporting ATPase 12 plasma membrane type	
NbS00021769g0027.1	13.19	1.45	-8.57	1.54705		
NbS00002551g0009.1	3.24	0.29	-8.52	1.23079		
NbS00011054g0001.1	7.00	0.74	-8.49	1.28183		
NbS00003513g0007.1	83.77	9.80	-8.47	2.84407		
NbS00014955g0018.1	5.15	0.53	-8.34	1.65275	Unknown protein	
NbS00006006g0011.1	29.26	3.45	-8.26	2.70325	Unknown protein	

NbS00005457g0204.1	32.88	3.94	-8.16	2.60062		
NbS00052331g0005.1	9.74	1.13	-8.01	2.16241	Glutamine amidotransferase YLR126C	
NbS00038068g0013.1	4.13	0.43	-8.01	1.91174	Vacuolar cation/proton exchanger 5	
NbS00015208g0005.1	3.44	0.34	-8.00	1.07012	WRKY transcription factor 16	
NbS00025445g0003.1	1.82	0.14	-7.89	1.44299	Beta carotene hydroxylase 2	
NbS00004485g0001.1	11.76	1.48	-7.49	1.4425	Calcium binding protein PBP1	
NbS00016881g0014.1	3.38	0.37	-7.37	1.54705	Core 2/I branching beta 1 6 N acetylglucosaminyltransferase	
NbS00028927g0001.1	4.35	0.51	-7.24	1.09298	Auxin induced SAUR protein	
NbS00005386g0003.1	120.42	16.62	-7.21	2.62386	BON1 associated protein 2	
NbS00025385g0005.1	518.62	72.04	-7.19	2.85803	Cathepsin B cysteine proteinase 5	
NbS00042475g0001.1	77.86	10.87	-7.11	2.52592	BON1 associated protein 2	
NbS00014697g0001.1	6.70	0.86	-7.11	1.30951	SKP1 protein 1	
NbS00004569g0028.1	24.39	3.37	-7.05	2.50497	Phosphoglycerate mutase	
NbS00033675g0003.1	34.77	4.89	-6.99	2.34157	Calcium binding protein CML45	
NbS00019421g0014.1	68.76	9.89	-6.89	2.5536	Cysteine rich repeat secretory protein 12	
NbS00031545g0001.1	26.54	3.81	-6.81	2.25933	Soybean gene regulated by cold 2	
NbS00028900g0005.1	14.38	2.05	-6.72	2.29882	Cathepsin B cysteine proteinase	
NbS00037868g0004.1	87.97	13.03	-6.70	2.54925	UPF0497 membrane protein 17	
NbS00007391g0001.1	47.78	7.11	-6.64	2.19524		
NbS00032991g0003.1	50.56	7.55	-6.62	2.3864	Soybean gene regulated by cold 2	
NbS00034618g0001.1	5.29	0.72	-6.61	1.07576	Unknown protein	
NbS00004020g0003.1	5.91	0.81	-6.61	1.81199	Beta carotene hydroxylase 2	
NbS00004868g0004.1	27.03	4.02	-6.58	1.90706	Unknown protein	
NbS00021769g0011.1	18.13	2.71	-6.49	2.02864		
NbS00016628g0001.1	8.76	1.30	-6.33	1.35222	Hypothetical protein	
NbS00027430g0009.1	13.57	2.07	-6.30	2.03865	RING H2 finger protein ATL22	
NbS00009695g0005.1	1.10	0.09	-6.17	1.30499	Transmembrane 9 member 4	
NbS00005203g0009.1	2.81	0.38	-6.06	1.33335	Alternative oxidase mitochondrial	
NbS00018731g0020.1	1.28	0.13	-5.97	1.51983	Calcium transporting ATPase 13 plasma membrane type	

NbS00047839g0003.1	8.32	1.34	-5.86	1.91897	Beta carotene hydroxylase 2	
NbS00006310g0007.1	4.02	0.61	-5.84	1.25359	ATP dependent RNA helicase eIF4A	
NbS00007436g0003.1	11.33	1.86	-5.84	1.70332	Sterile alpha motif SAM domain containing protein	
NbS00038702g0003.1	6.01	0.95	-5.83	1.08714	Hypothetical protein VITISV_009284	
NbS00000634g0108.1	1.70	0.21	-5.81	1.02422		
NbS00024411g0009.1	2560.6 8	447.16	-5.73	2.58612	Endochitinase Chitinase	
NbS00018281g0007.1	12.41	2.09	-5.72	1.15331		
NbS00026817g0018.1	10.43	1.76	-5.68	1.95974	Uncharacterized protein LOC100853376	
NbS00006309g0017.1	10.82	1.86	-5.57	1.95386		
NbS00022684g0002.1	10.02	1.72	-5.55	1.55504	Unknown protein	
NbS00011363g0101.1	11.20	1.95	-5.51	1.0188		
NbS00045861g0002.1	71.77	13.11	-5.44	2.18196	Unknown protein	
NbS00012621g0108.1	37.74	6.87	-5.43	2.00347		
NbS00024669g0003.1	29.34	5.36	-5.39	2.08095	Acetyl coenzyme A cis 3 hexen 1 ol acetyl transferase	
NbS00035332g0014.1	6.33	1.10	-5.34	1.0188	Basic helix loop helix	
NbS00028303g0001.1	7.63	1.35	-5.32	1.68147	Ankyrin repeat containing protein	
NbS00011648g0011.1	16.07	2.96	-5.29	2.21667	ARGONAUTE 1	
NbS00001236g0045.1	12.10	2.22	-5.26	2.07921	Subtilisin protease	
NbS00009990g0011.1	7.76	1.39	-5.26	1.66223	Copper ion binding protein	
NbS00045727g0001.1	142.44	27.26	-5.21	2.23126	Disease resistance response	
NbS00026127g0018.1	87.11	16.75	-5.18	2.15418	NAC domain transcription factor	
NbS00053630g0004.1	161.08	31.23	-5.15	2.36766	Acetyl coenzyme A cis 3 hexen 1 ol acetyl transferase	
NbS00055998g0002.1	70.61	13.66	-5.14	2.05891	Unknown protein	
NbS00025974g0007.1	14.75	2.89	-4.97	1.81956	Unknown protein	
NbS00017742g0008.1	7.68	1.47	-4.96	1.90637	NAC domain protein	
NbS00061402g0001.1	95.80	19.37	-4.93	2.07745	Copper transporter	
NbS00002289g0008.1	22.27	4.47	-4.90	2.10636	Vacuolar sorting receptor 7	
NbS00033080g0001.1	16.17	3.23	-4.88	1.49837	Unknown protein	
NbS00016343g0003.1	842.55	172.87	-4.87	2.29456	Glycine rich protein	
NbS00009224g0003.1	8.29	1.64	-4.84	1.65217	Fasciclin arabinogalactan protein 18	
NbS00031370g0112.1	2.98	0.54	-4.81	1.10051		
NbS00030820g0001.1	12.37	2.52	-4.76	1.42449	Unknown protein	

NbS00022638g0012.1	3.08	0.57	-4.74	1.70449		
NbS00022787g0012.1	16.12	3.33	-4.73	1.7173	HVA22 protein i	
NbS00061325g0006.1	0.81	0.09	-4.71	1.33882	LRR receptor serine/threonine protein kinase	
NbS00055076g0001.1	124.39	26.36	-4.70	1.98795	Unknown protein	
NbS00011088g0001.1	48.85	10.31	-4.70	1.87825	Calcium binding protein CML44	
NbS00015242g0008.1	7.03	1.42	-4.70	1.38318	LOB domain protein 25	
NbS00029024g0002.1	95.88	20.37	-4.69	1.90567	Uncharacterized protein LOC100810725	
NbS00054976g0001.1	17.82	3.73	-4.67	1.69446	Calcium binding protein CML30	
NbS00002267g0005.1	7.80	1.59	-4.67	1.18797	Unknown protein	
NbS00047968g0010.1	10.95	2.27	-4.66	1.72198	RNA recognition motif containing protein	
NbS00030233g0002.1	3.80	0.74	-4.62	1.35319	Receptor protein kinase	
NbS00036825g0003.1	57.00	12.26	-4.62	2.03634	E3 ubiquitin protein ligase RNF217	
NbS00004056g0005.1	167.55	36.42	-4.59	2.10171	Glutaredoxin C9	
NbS00001433g0005.1	147.33	32.11	-4.58	1.96798		
NbS00044915g0003.1	54.63	11.86	-4.58	2.08695	Squalene monooxygenase	
NbS00001177g0011.1	17.17	3.71	-4.54	1.92388	Vacuolar iron transporter VIT	
NbS00025701g0011.1	2.82	0.54	-4.52	1.66127	ARGONAUTE 1	
NbS00035332g0011.1	6.21	1.31	-4.49	1.01433	Basic helix loop helix	
NbS00010196g0001.1	10.00	2.15	-4.49	1.60377	Unknown protein	
NbS00007113g0017.1	1.74	0.31	-4.48	1.27536	WD 40 repeat	
NbS00008798g0006.1	1.58	0.28	-4.48	1.0188	Aspartate aminotransferase	
NbC25742659g0003.1	2482.74	557.65	-4.45	2.22705	Glucan endo 1 3 beta glucosidase%2C acidic isoform GI9	
NbS00035466g0005.1	76.97	17.35	-4.42	1.98208	Cysteine rich repeat secretory protein 12	
NbS00010129g0001.1	2385.81	541.78	-4.40	2.20982	Beta 1 3 glucanase	
NbS00020273g0011.1	254.02	57.67	-4.40	2.04108	Pathogenesis related protein PR 4B	
NbS00028343g0017.1	42.53	9.63	-4.38	1.97157	Ribose phosphate pyrophosphokinase 4	
NbS00006199g0004.1	4.20	0.90	-4.30	1.45466	Inorganic phosphate transporter	
NbS00007836g0001.1	625.86	146.44	-4.27	2.11943	Strictosidine synthase	

NbS00024261g0008.1	5.47	1.21	-4.24	1.33809	Ribose phosphate pyrophosphokinase 4	
NbS00026420g0026.1	49.46	11.60	-4.24	1.97122	F box	
NbS00010812g0004.1	12.79	2.95	-4.22	1.65383	F box protein PP2 B10	
NbS00016708g0006.1	5.55	1.24	-4.20	1.66093	Ubiquitin conjugating enzyme 23	
NbS00036148g0004.1	37.34	8.81	-4.20	1.80753	WRKY transcription factor 9	
NbS00019157g0035.1	11.93	2.78	-4.18	1.57534	Serine/threonine protein phosphatase 2C	
NbS00043015g0001.1	61.06	14.62	-4.15	1.87155	Heavy metal associated domain containing protein expressed	
NbS00054521g0001.1	13.99	3.31	-4.13	1.72412	MATE efflux DTX1	
NbS00023392g0005.1	6.78	1.57	-4.12	1.58656	Pectinesterase/pectinesterase inhibitor 25	
NbS00003323g0003.1	22.21	5.33	-4.11	1.57567	Ribosomal protein alanine N acetyltransferase	
NbS00001559g0013.1	40.69	9.83	-4.11	1.76128	Armadillo/beta catenin repeat	
NbS00059236g0004.1	14.71	3.53	-4.08	1.72663	Squalene monooxygenase	
NbS00031402g0006.1	14.07	3.39	-4.06	1.10196		
NbS00024147g0002.1	14.40	3.50	-4.02	1.60117	ATP binding protein	
NbS00014993g0001.1	9.78	2.36	-4.02	1.16406		
NbS00036311g0001.1	40.70	10.07	-4.01	1.5432		
NbS00014120g0117.1	194.65	47.50	-4.09	1.99793		

Table S2. Oligonucleotides used in this study

No.	Name	Sequence (from 5' to 3’)	Usage
1	CRN115-F	cgcggatccATGTTCCCAGTGGACATCGATGG	Insertion of *PsCRN115* without SP into pBinGFP2
2	CRN115-R	gctctagaTCAGCTGAAATAACTAAACG	
3	NPTII-F	TGACTGGGCACAACAGACAAT	Primers for *NPTII*, used for genomic PCR assay
4	NPTII-R	AAGACCCCAACGAGAAGC	
5	EF1α-F	AGAGGCCCTCAGACAAAC	Primers of *NbEF1α*, used for real time PCR assay
6	EF1α-R	TAGGTCCAAAGGTCACAA	
7	PR1b-F	GTGGACACTATACTCAGGTG	Primers of *NbPR1b*, used for real time PCR assay
8	PR1b-R	TCCAACTTGGAATCAAAGGG	
9	PR2b-F	AGGTGTTTGCTATGGAATGC	Primers of *NbPR2b*, used for real time PCR assay
10	PR2b-R	TCTGTACCCACCATCTTGC	
11	RbohA-F	GACTCGTTCCAGCGCTCATA	Primers of *NbRbohA*, used for real time PCR assay
12	RbohA-R	TGTGCGAAATCGGAACGGTA	
13	RbohB-F	TCACAAGAGCTCAGGCGTTT	Primers of *NbRbohB*, used for real time PCR assay
14	RbohB-R	TCATCGAACCGCTTCTCGAC	
15	NbS00034783 g0004.1-F	ATGACAATGTGCTGCTGA	Primers for real time PCR assay
16	NbS00034783 g0004.1-R	TTTTGGGTTTCTTAGGCT	
17	NbC25233207 g0001.1-F	ATGAGAAGATTCAGGCTCC	Primers for real time PCR assay
18	NbC25233207 g0001.1-R	GAACAGTTACAGTAAGCACTC	
19	NbS00009579 g0009.1-F	ATTGGGCTTTCCAGGTTC	Primers for real time PCR assay
20	NbS00009579 g0009.1-R	TGCCACGTATCATTCTTCTC	
21	NbC25673618 g0001.1-F	ATTCAGTCATTTTATCCCAAGA	Primers for real time PCR assay
22	NbC25673618 g0001.1-R	TTACAAGCCCTGCCTACA	
23	NbC24805505 g0002.1-F	TTTTGGCAGTGGAAGAAG	Primers for real time

24	NbC24805505 g0002.1-R	AGAGCAATAGTGCTAGACAGAG	PCR assay
25	NbS00038435 g0004.1-F	TTGGTATTCGTAAGGGAC	Primers for real time PCR assay
26	NbS00038435 g0004.1-R	TAGGGGAGTAGATTTTGG	
